# Integrated genomic analysis of *Stenotrophomonas maltophilia*: resistome, virulence-associated genes, predicted mobile genetic elements, and core-genome phylogeny

**DOI:** 10.3389/fmicb.2026.1880458

**Published:** 2026-09-09

**Authors:** Rong Sun, Yandan Liu, Xiaoli Cao, Chang Liu, Shuo Gao, Bingbo Zhou, Qianjin Sun

**Affiliations:** 1 Department of Clinical Laboratory, Xuzhou Hospital of Traditional Chinese Medicine, Xuzhou, Jiangsu, China; 2 Department of Clinical Laboratory, Tianjin Academy of Traditional Chinese Medicine Affiliated Hospital, Tianjin, China; 3 Department of Laboratory Medicine, Nanjing Drum Tower Hospital, The Affiliated Hospital of Nanjing University Medical School, Nanjing, Jiangsu, China; 4 The Center of Medicine Genetic in Gansu Provincial Maternity and Child-Care Hospital (Gansu Provincial Central Hospital), Gansu Provincial Clinical Research Center for Birth Defects and Rare Diseases, Lanzhou, China

**Keywords:** antimicrobial resistance, mobile genetic elements, multi-locus sequence typing, *Stenotrophomonas maltophilia*, virulence factors

## Abstract

**Background:**

*Stenotrophomonas maltophilia* is an emerging opportunistic pathogen characterized by intrinsic multidrug resistance and broad ecological distribution. However, its genomic characteristics across publicly available clinical, environmental, and animal isolates remain incompletely defined.

**Objective:**

This study aimed to characterize the taxonomic distribution, antimicrobial resistance genes (ARGs), virulence-associated genes (VAGs), sequence types (STs), predicted mobile genetic elements (MGEs), orthogroup-occupancy patterns, and core-genome sequence variation of publicly available *S. maltophilia* isolates.

**Methods:**

A total of 3,343 high-quality *Stenotrophomonas* candidate genomes obtained from NCBI were subjected to average nucleotide identity (ANI)-based taxonomic verification using the NCBI Prokaryotic Genome Annotation Pipeline. Of these, 1,584 genomes unambiguously assigned to *S. maltophilia* were retained for genomic analyses. ARGs were identified using a custom BLASTN-based nucleotide screen validated against AMRFinderPlus, and VAGs, multi-locus sequence types (MLST), and predicted MGEs were characterized using complementary bioinformatic approaches. Source-stratified analyses used the 1,054 genomes with resolved isolation-source metadata. Pangenome composition and lineage-specific core-genome phylogenetic relationships were evaluated separately for 95 ST4 and 113 ST5 genomes.

**Results:**

*S. maltophilia* represented 1,584 of the 3,343 candidate genomes (47.4%). Country-level metadata were resolved for 1,044 genomes, representing 35 countries, whereas 540 had missing or unresolved geographic information. Among the 1,054 source-resolved genomes, 856 (81.2%) were human-derived, 154 (14.6%) were environmental, and 44 (4.2%) were animal-derived. The custom ARG screen identified 12,215 loci representing 67 ARG designations, while 38 non-redundant VAGs were detected. The efflux-associated genes *emrA*, *emrB*, *emrC*, and *smeF*, together with the intrinsic β-lactamase gene *blaL1*, were widely distributed, whereas acquired carbapenemase genes were uncommon. Source-associated differences were limited to selected ARGs and VAGs rather than broad changes in ARG or VAG burden. Among 1,388 typeable genomes, 234 STs were identified, with ST5, ST4, ST31, ST162, and ST115 being the most frequently represented. A total of 1,140 predicted ARG–MGE co-localization events were identified; predicted plasmid-associated contigs represented the largest category of ARG–MGE co-localization events, accounting for 898 events (78.8%), although these computational assignments did not establish confirmed plasmid carriage or transferability. In the pangenome comparison, ST4 showed a larger rare-gene and strain-specific orthogroup component, whereas ST5 showed greater core-genome sequence separation according to retained variable-site counts and pairwise SNP-distance metrics. ST4 had higher proportions of strain-specific orthogroups (3.4% versus 1.3%) and unassigned genes (2.7% versus 0.8%), whereas the shell-plus-cloud proportions were similar between ST4 and ST5 (46.7% versus 46.2%). ST5 had a higher orthogroup-assignment rate (99.2% versus 97.3%) and a higher proportion of orthogroups meeting the ≥ 99% core threshold (52.4% versus 49.8%). Core-genome analyses showed a higher pre-recombination variable-site density in ST5 than in ST4 (4.54 versus 3.50 sites/kb), more retained variable sites after recombination filtering (3,269 versus 2,295), and a higher median pairwise SNP distance (127 versus 108 SNPs).

**Conclusion:**

This analysis of publicly available *S. maltophilia* genomes identified widely distributed intrinsic resistance determinants, uncommon acquired carbapenemases, selected source-associated gene differences, and frequent computational co-localization of ARGs with sequences predicted to be plasmid-associated. ST4 contained a larger strain-specific and unassigned-gene component, whereas ST5 showed greater core-genome sequence separation according to variable-site density and pairwise SNP-distance metrics. These findings support continued genomic surveillance across clinical, environmental, and animal sources while emphasizing the need for epidemiologically representative sampling, complete genome assemblies, and experimental validation of predicted ARG mobility.

## 1 Introduction

*Stenotrophomonas maltophilia* is an opportunistic Gram-negative bacterium recovered from a wide range of clinical, environmental, and animal-associated settings (Mojica et al., 2022; Mikhailovich et al., 2024). It has increasingly attracted clinical attention because of its intrinsic multidrug resistance, environmental adaptability, and capacity to cause infections, particularly in immunocompromised individuals and patients with underlying diseases (Brooke, 2021; Gibb and Wong, 2021). Although *S. maltophilia* is commonly regarded as a healthcare-associated opportunistic pathogen, it is also frequently detected in water, soil, plants, animals, and hospital environments. This broad ecological distribution suggests potential connections between environmental reservoirs and clinical settings, although the direction and frequency of such transmission cannot be inferred from genomic surveillance data alone (Li et al., 2023; Crisan and Goldberg, 2025).

The clinical importance of *S. maltophilia* is closely associated with its complex antimicrobial resistance profile. This species harbors intrinsic resistance mechanisms, including β-lactamases and multidrug efflux systems, and may also acquire additional resistance determinants through horizontal gene transfer (Delgarm Shams-Abadi et al., 2023). Genes associated with resistance to β-lactams, aminoglycosides, quinolones, sulfonamides, tetracyclines, and other antimicrobial classes have been reported, complicating therapeutic management (Vattanaviboon et al., 2026). In addition to antimicrobial resistance genes (ARGs), virulence-associated genes (VAGs) involved in adhesion, biofilm formation, effector delivery, motility, immune modulation, and stress survival may contribute to colonization, persistence, and pathogenicity (Mikhailovich et al., 2024). However, the global distribution of ARGs and VAGs across available genomes from different sources, geographic regions, and sequence types (STs) remain incompletely characterized (Gröschel et al., 2020; Li et al., 2023).

Whole-genome sequencing (WGS) provides a powerful framework for integrating taxonomic verification, ARG and VAG profiling, multilocus sequence typing (MLST), prediction of mobile genetic elements (MGEs), pangenome analysis, and phylogenetic reconstruction at large scale (Koonin et al., 2021). Previous genomic studies have described important features of *S. maltophilia* diversity and antimicrobial resistance, but many have examined geographically or epidemiologically restricted collections or have not integrated these analytical components within a single standardized dataset (Brooke, 2021; Kullar et al., 2022). In addition, analyses of public genome collections require careful control of taxonomic assignment, metadata completeness, analytical denominators, and the distinction between computational prediction and experimentally confirmed biological function. Questions therefore remain regarding the distribution of selected ARGs and VAGs among human, animal, and environmental isolates, the genomic characteristics of frequently represented sequence types (STs), and the relative patterns of gene-content and core-genome sequence variation within major lineages (Li et al., 2023).

In this study, we collected publicly available *Stenotrophomonas* genome assemblies and raw sequencing datasets from NCBI and applied standardized genome assembly, quality control, annotation, and average nucleotide identity (ANI)-based taxonomic verification. Of 3,343 high-quality candidate genomes, 1,584 were assigned unambiguously to *S. maltophilia* and retained for further analyses. We characterized ARGs, VAGs, MLST, and predicted mobile genetic elements (MGEs) and evaluated source-associated differences using the subset with resolved isolation-source metadata. We also compared resistance-gene and virulence-gene profiles among frequently represented STs, assessed predicted contig-level ARG–MGE co-localization, examined orthogroup-occupancy patterns in ST4 and ST5, and reconstructed lineage-specific core-genome phylogenies (Kullar et al., 2022). By distinguishing gene-content variation, core-genome sequence separation, and computationally predicted MGEs associations, this study provides an integrated genomic characterization of the available public *S. maltophilia* collection while recognizing the limitations of uneven sampling, incomplete metadata, fragmented assemblies, and prediction-based analyses.

## 2 Materials and methods

### 2.1 Genome collection, assembly, quality control, and taxonomic classification

**FIGURE 1:**
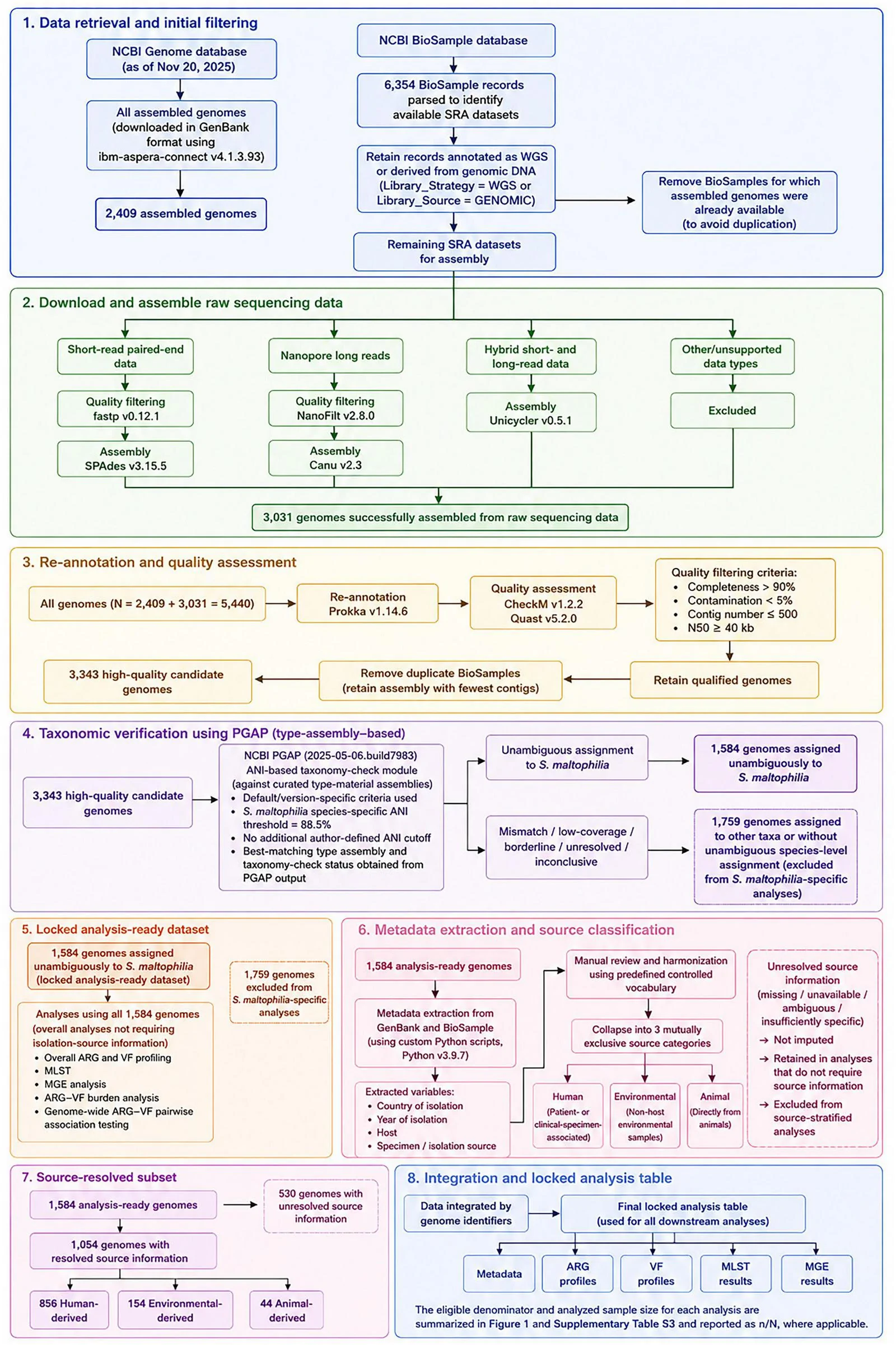
Workflow for genome collection, quality control, taxonomic validation, and construction of the *Stenotrophomonas maltophilia* analysis-ready genome dataset. NCBI, National Center for Biotechnology Information; SRA, Sequence Read Archive; WGS, whole-genome sequencing; DNA, deoxyribonucleic acid; PGAP, Prokaryotic Genome Annotation Pipeline; ANI, average nucleotide identity; ARG, antimicrobial resistance gene; VF, virulence factor; MLST, multilocus sequence typing; MGE, mobile genetic element.

Genome data of *Stenotrophomonas* spp. were collected from the National Center for Biotechnology Information (NCBI) databases following the workflow summarized in Figure 1. As of November 20, 2025, all available assembled genomes were retrieved from the NCBI Genome database and downloaded in GenBank format using ibm-aspera-connect v4.1.3.93, resulting in 2,409 assembled genomes. In parallel, 6,354 BioSample records were obtained from the NCBI BioSample database and screened to identify available Sequence Read Archive (SRA) datasets. Records annotated as WGS data or derived from genomic DNA were retained according to SRA metadata criteria (Library_Strategy = WGS or Library_Source = GENOMIC). BioSamples for which assembled genomes were already available were removed to avoid duplication.

The remaining SRA datasets were downloaded and assembled according to sequencing type. Short-read paired-end data were quality-filtered using fastp v0.12.1 and assembled with SPAdes v3.15.5; Nanopore reads were filtered using NanoFilt v2.8.0, long-read-only datasets were assembled using Canu v2.3; whereas hybrid short- and long-read datasets were assembled using Unicycler v0.5.1. In total, 3,031 genomes assemblies were reconstructed from raw sequencing data. To minimize annotation bias arising from heterogeneous genome annotation pipelines, all genome assemblies were uniformly re-annotated using Prokka v1.14.6 (Seemann, 2014). Genome quality was assessed using CheckM v1.2.2 and Quast v5.2.0 (Gurevich et al., 2013; Parks et al., 2015). Genomes with completeness > 90%, contamination < 5%, contig number ≤ 500, and N50 ≥ 40 kb were retained. Duplicate BioSamples were resolved by retaining the assembly with the fewest contigs. Following quality filtering and deduplication, 3,343 high-quality candidate genomes were subjected to species-level taxonomic verification.

Taxonomic identification was performed using a local installation of the NCBI Prokaryotic Genome Annotation Pipeline (PGAP, version 2025-05-06.build7983) with the integrated ANI-based taxonomy-check module (Tatusova et al., 2016; Ciufo et al., 2018). This procedure represented type-assembly-based taxonomic verification rather than de novo ANI clustering or species delimitation. The version-specific default criteria implemented in PGAP were used without modification. PGAP default taxonomy-verification criteria were applied without modification. For *S. maltophilia* (NCBI Taxonomy ID: 40324), the species-specific ANI threshold implemented in the PGAP framework was 88.5%; no additional author-defined ANI cutoff was applied.

Each query genome was compared against curated type-material assemblies included in the PGAP taxonomy database. The best-matching type assembly and taxonomy-check status were obtained directly from PGAP output based on ANI values, query/reference alignment fractions, and version-specific taxonomy-verification rules. No manual reference genome selection, post hoc reassignment, or additional tie-breaking procedures were performed. Genomes with unambiguous assignment to *S. maltophilia* were retained, whereas genomes with mismatch, low-coverage, borderline, unresolved, or otherwise inconclusive taxonomy-check results were excluded from *S. maltophilia*-specific analyses. Genome-level taxonomic verification results and inclusion/exclusion outcomes are provided in Supplementary Table S1.

### 2.2 Cohort definition, metadata harmonization, and analytic denominators

The 1,584 genomes assigned unambiguously to *S. maltophilia* constituted the locked analysis-ready dataset (Figure 1). Analyses that did not require isolation-source information, including overall ARG and VAG profiling, MLST, MGE analysis, ARG–VAG burden analysis, and genome-wide ARG–VAG pairwise association testing, were performed using all 1,584 genomes.

Strain metadata were extracted from the corresponding GenBank and BioSample records using custom Python scripts implemented in Python v3.9.7. Extracted variables included country of isolation, year of isolation, host, and specimen or isolation source. Isolation-source metadata were manually reviewed and harmonized using a predefined controlled vocabulary. Records with sufficient source information were manually reviewed and harmonized into three biological source categories: human-derived, environmental, and animal-derived. Records lacking sufficient information for reliable classification were assigned as unresolved and excluded from source-stratified analyses. Detailed category-collapsing rules and mappings of original source terms are provided in Supplementary Table S2.

The analytical denominator was defined according to the availability of relevant metadata for each analysis. Source-stratified analyses were restricted to genomes with resolved isolation-source information, whereas analyses not requiring source information included all genomes in the locked analysis-ready dataset. Metadata, ARG profiles, VAG profiles, MLST results, and MGE results were integrated using genome identifiers into a single locked analysis table used for all downstream analyses. The analytical denominators for individual analyses are summarized in Supplementary Table S3.

### 2.3 Identification of ARGs and validation of the ARG screening workflow

ARGs were identified using a custom nucleotide-based screening workflow, and the concordance of this approach was independently evaluated using AMRFinderPlus. The primary ARG dataset used for downstream analyses was generated using a custom BLASTN-based nucleotide-screening workflow against curated ARG reference sequences from the National Center for Biotechnology Information (NCBI) Pathogen Detection Reference Gene Catalog (release 2025-12-03.1; accessed December 3, 2025). The complete versioned reference sequence set used for ARG screening, including reference accession numbers, gene symbols, resistance classes, and database metadata, is provided in Supplementary Table S4.

Coding sequences predicted by Prokka v1.14.6 (Seemann, 2014) were used as nucleotide queries and searched against ARG reference sequences using BLASTN v2.13.0. Candidate matches were initially retained using the following criteria: E-value ≤ 1 × 10^−5^, nucleotide identity ≥ 90%, and an alignment length ≥ 30 bp. Query coverage was calculated as the proportion of the Prokka-predicted coding sequence covered by the BLASTN alignment, whereas subject coverage was calculated as the proportion of the complete reference ARG sequence covered by the alignment. A final ARG assignment required nucleotide identity ≥ 90%, query coverage ≥ 90%, and subject/reference-sequence coverage ≥ 90%. The ≥ 30-bp alignment-length threshold was applied only as an initial screening criterion to exclude short non-informative alignments and was not considered sufficient evidence for ARG assignment. Matches representing partial or truncated sequences were excluded when they failed the predefined query coverage or subject coverage thresholds and were not included in the primary ARG presence/absence dataset.

Multiple BLASTN hits were resolved at the genomic-locus level. Overlapping hits corresponding to the same coding sequence or genomic locus were merged, and the best-supported assignment was selected according to bit score, followed by nucleotide identity, subject coverage, and query coverage. Multiple detections of the same resistance gene at an identical genomic locus were counted as a single ARG locus, whereas non-overlapping hits located at distinct genomic positions were retained as independent ARG loci. For loci matching multiple allelic reference sequences, the best-supported allele was selected using the same ranking criteria. When allele-level discrimination was not supported by the available sequence evidence, assignments were reported at the gene or gene-family level rather than being arbitrarily assigned to a specific allele.

To evaluate the reliability and consistency of the custom BLASTN-based ARG screening workflow, ARG predictions were independently compared with AMRFinderPlus v2.4.5 across all 1,584 *S. maltophilia* genomes. AMRFinderPlus analyses were performed using Prokka-derived protein predictions, and the input mode, command-line parameters, and database version are provided in Supplementary Table S5. Because AMRFinderPlus reports both antimicrobial resistance determinants and stress-response-associated genes, validation analyses were restricted to records classified as Type = AMR; records classified as STRESS were excluded from ARG comparisons.

Agreement between the two approaches was evaluated at the locus level using shared Prokka-derived identifiers. Exact identifier matches between BLASTN-derived ARG calls and AMRFinderPlus AMR records were considered concordant loci. ARG loci detected exclusively by the custom BLASTN workflow or exclusively by AMRFinderPlus were recorded separately. Gene symbols and resistance classifications were further compared among matched loci to identify differences caused by allele nomenclature or annotation conventions. The comparison was conducted bidirectionally to evaluate both the recovery of BLASTN-derived ARGs by AMRFinderPlus and additional AMRFinderPlus-specific detections.

AMRFinderPlus was used as an independent validation framework and did not replace the custom BLASTN-derived ARG dataset used for downstream analyses. Because the two approaches are based on different detection principles, the comparison was interpreted as an assessment of concordance between analytical frameworks rather than an estimate of diagnostic sensitivity or specificity. Detailed results of the concordance analysis between the custom BLASTN workflow and AMRFinderPlus are provided in Supplementary Table S5.

### 2.4 Identification of VAGs

VAGs were identified using a custom BLASTN-based nucleotide screening workflow against DNA sequences from the VFDB core dataset downloaded from the Virulence Factor Database (VFDB)^1^ on April 10, 2026. Because VFDB is continuously updated, the database used in this study was defined by the exact dataset type and download date rather than by the current content displayed on the VFDB website. The complete reference sequence set used for VAG screening, including VFDB identifiers, gene symbols, and reference accession information, is provided in Supplementary Table S6.

Coding sequences predicted by Prokka v1.14.6 (Seemann, 2014) were extracted from the *S. maltophilia* genomes and searched against the VFDB reference sequence set using BLASTN v2.13.0. Candidate hits were retained when they met the following criteria: E-value ≤ 1 × 10^−5^, nucleotide identity ≥ 90%, query coverage ≥ 90%, subject coverage ≥ 90%, and an initial alignment-length filter of ≥ 30 bp. The alignment-length threshold was used only as an initial filtering step to remove short non-informative matches and was not considered sufficient evidence for VAG assignment. Hits failing the final identity or coverage requirements were excluded to minimize false-positive VAG assignments (Cahill et al., 2022).

Because multiple VFDB reference sequences may correspond to the same VAG, redundant reference sequences were collapsed at the gene-symbol level before downstream analyses. Multiple hits representing the same VAG within an individual genome were counted once for VAG presence/absence and VAG burden analyses. When multiple reference sequences mapped to the same gene symbol, the gene-level assignment was retained rather than treating individual VFDB reference entries as separate virulence determinants.

### 2.5 Multi-locus sequence typing

MLST was performed using the *S. maltophilia* MLST scheme curated in PubMLST.^2^ The allele-sequence definitions and MLST profile table used in this study were downloaded on April 10, 2026, and this downloaded snapshot was used consistently throughout the analysis. Because the PubMLST typing database is continuously curated and updated, the database snapshot is identified by the scheme name and exact download date. The corresponding allele and profile files used for analysis are documented in the reproducibility information.

The seven housekeeping gene loci (*atpD, gapA, guaA, mutM, nuoD, ppsA, and recA*) were identified using MLST software v2.23.0, and the resulting allele sequences were compared with the allele definitions and allelic profiles contained in the downloaded PubMLST snapshot. Allele numbers and STs were assigned according to the allele and profile definitions available in the April 10, 2026 snapshot. Allelic combinations not represented in the downloaded profile table were recorded as unassigned/putative novel profiles unless a formal ST designation had been assigned by PubMLST.

### 2.6 Identification of mobile genetic elements and predicted ARG–MGE associations

MGEs were identified in the 1,584 *S. maltophilia* genomes using complementary bioinformatic approaches. Plasmid-associated contigs were predicted using geNomad v1.12.0 and MOB-suite v3.1.9 (Robertson and Nash, 2018; Camargo et al., 2024). Bacteriophage and prophage regions were detected using PHASTEST v1.0.1 (Wishart et al., 2023), integrative conjugative elements (ICEs) and integrative mobilizable elements (IMEs) using ICEfinder v2.0 (Liu et al., 2019; Camargo et al., 2024), integrons using IntegronFinder v2.0 (Cury et al., 2016), insertion sequences (ISs) using ISEScan v1.7.2.3 (Xie and Tang, 2017), and additional mobile elements using MobileElementFinder v1.0.3 (Johansson et al., 2021). Unless otherwise specified, all tools were run using default parameters without manual modification of prediction thresholds. Software versions, database releases, access dates, and key parameters are summarized in Supplementary Table S7.

Plasmid predictions were treated as computational contig-level classifications of plasmid-associated sequences rather than experimentally confirmed plasmid localization. For geNomad, the default post-classification criteria were applied, including the default plasmid score threshold of ≥ 0.7. MOB-suite plasmid/chromosome classifications were obtained from contig-level contig_report.txt files generated by MOB-recon under default settings. The MOB-suite outputs from all genomes were merged using unique genome–contig identifiers to prevent duplication or ambiguity between contigs with identical names in different assemblies.

A broad plasmid-associated prediction set was defined using a union approach: a contig was classified as predicted plasmid-associated when either geNomad or MOB-suite assigned it as plasmid-associated. Predictions supported by both tools were additionally classified as high-confidence plasmid-associated sequences. Contigs classified as plasmid-associated by only one tool were retained in the broad prediction set but were designated as single-tool-supported predictions rather than high-confidence plasmid-associated sequences. Concordant and discordant classifications were recorded separately in the contig-level evidence table.

Predicted ARG–MGE associations were evaluated at the contig and coordinate levels. For plasmid-associated sequences, an ARG was considered associated with a predicted plasmid when the ARG coordinates were located on the same contig classified as plasmid-associated according to the criteria described above. For MGEs predicted as defined genomic intervals, including prophages, ICEs, IMEs, integrons, and ISs, an association was recorded only when the ARG coordinates overlapped the predicted MGE coordinates on the same contig. Co-occurrence of an ARG and an MGE prediction in the same genome without same-contig localization and coordinate overlap was not considered evidence of association. Associations were not inferred across contig boundaries, and contig breaks in fragmented assemblies were treated as unresolved sequence boundaries.

Assembly contiguity was considered when interpreting ARG–MGE associations. Of the 1,584 genomes, 69 were represented by a single contig and were analyzed as a higher-contiguity subset, whereas the remaining 1,515 multi-contig genomes were classified as fragmented draft assemblies. Single-contig status was not assumed to indicate long-read sequencing or confirmed complete-genome closure when sequencing-platform or closure metadata were unavailable. ARG–MGE predictions were summarized for the complete dataset and separately according to assembly-contiguity category to assess the potential influence of assembly fragmentation.

A contig-level evidence table was generated containing the genome identifier, assembly-contiguity category, contig identifier and length, ARG identity and coordinates, predicted MGE category and coordinates, prediction tool, plasmid-classification score or status where available, agreement between geNomad and MOB-suite, and evidence-confidence category. All reported ARG–MGE relationships therefore represent predicted sequence-level co-localization. They do not establish that an ARG is located on a complete plasmid or intact mobile element, nor do they demonstrate mobilization, transferability, or horizontal transmission.

### 2.7 Orthogroup-based pangenome characterization of ST4 and ST5 lineages

Pangenome analysis was performed separately for the 95 ST4 and 113 ST5 *S. maltophilia* genomes to characterize lineage-specific orthogroup occupancy and gene-assignment patterns. Predicted protein sequences from each lineage were analyzed using OrthoFinder v2.5.5 (Emms and Kelly, 2019) with default parameters to identify orthologous gene groups.

Orthogroups were classified using predefined genome-occupancy thresholds applied independently to each lineage. Strict-core orthogroups were defined as orthogroups present in 100% of genomes within a lineage (Gröschel et al., 2020). Near-core orthogroups were defined as orthogroups present in ≥ 99% but < 100% of genomes. Shell orthogroups were present in ≥ 15% but < 99% of genomes. Cloud orthogroups were present in > 1 but < 15% of genomes. Orthogroups detected in exactly one genome were classified as strain-specific orthogroups (McDaniel et al., 2023). These categories were mutually exclusive, and their sum equaled the total number of observed orthogroups within each lineage.

Because genome occupancy is represented by integer counts, the same percentage thresholds resulted in lineage-specific integer cutoffs. For ST4, the ≥ 99% near-core threshold required presence in all 95 genomes because 94/95 genomes corresponded to 98.95%, below the predefined threshold; shell orthogroups were present in 15–94 genomes, cloud orthogroups in 2–14 genomes, and strain-specific orthogroups in one genome. For ST5, strict-core orthogroups were present in 113 of 113 genomes, near-core orthogroups in 112 of 113 genomes, shell orthogroups in 17–111 genomes, cloud orthogroups in 2–16 genomes, and strain-specific orthogroups in one genome.

The term accessory genome was used only as an umbrella term for all non-core orthogroups, comprising shell, cloud, and strain-specific orthogroups. When orthogroups present in more than one genome but below the ≥ 99% core threshold were reported separately, they were explicitly described as shell-plus-cloud orthogroups rather than as the complete accessory genome. Unassigned genes were reported separately and were not included in the orthogroup-category totals.

For each lineage, the following metrics were extracted or calculated from the OrthoFinder V2.5.5 output: total number of predicted genes, number and percentage of genes assigned to orthogroups, number and percentage of unassigned genes, total number of observed orthogroups, and the numbers and proportions of strict-core, near-core, shell, cloud, and strain-specific orthogroups. The number of genes assigned to strain-specific orthogroups and the number of single-copy orthogroups were also recorded. Orthogroup-occupancy distributions were generated across all possible genome counts to verify category assignment and ensure that the mutually exclusive categories summed to the total observed orthogroup count.

No pangenome-accumulation curve, Heaps’ law model, openness parameter, or other formal open-versus-closed pangenome analysis was fitted. Therefore, the observed orthogroup counts were not used to classify either ST4 or ST5 as having an open or closed pangenome. Because the two lineage datasets contained different numbers of genomes, cumulative quantities, including total gene and orthogroup counts, were considered sample-size-sensitive. Comparisons were consequently restricted to explicitly defined occupancy and gene-assignment metrics. Counts and proportions were treated as descriptive summaries of the sampled lineage datasets and were not interpreted as estimates of lineage-wide pangenome openness.

### 2.8 Lineage-specific phylogenetic and SNP-distance analysis

Core-genome SNP-based phylogenetic analysis was performed separately for ST4 and ST5 *S. maltophilia* strains. All 95 ST4 and 113 ST5 genomes that met the genome-quality, taxonomic-verification, and MLST criteria were included, and no additional genomes were excluded after lineage assignment. Phylogenetic reconstruction was based on concatenated nucleotide alignments of single-copy core orthogroups. Orthogroup identification was performed independently for ST4 and ST5 using OrthoFinder v2.5.5 (Emms and Kelly, 2019). Only single-copy core orthogroups shared by all genomes within each lineage were retained for phylogenetic reconstruction, thereby excluding multicopy orthogroups and potentially ambiguous paralogous loci. Corresponding nucleotide sequences of retained orthogroups were extracted, aligned using MUSCLE v3.8.1551, and concatenated according to a consistent orthogroup order across genomes. The resulting concatenated alignments comprised 1,379,917 bp for ST4 and 1,853,398 bp for ST5. No additional repeat masking was applied before recombination analysis because recombinant regions were subsequently assessed using ClonalFrameML v1.12.

Variable nucleotide positions were identified from the concatenated alignments using SNP-sites v2.5.1 (Page et al., 2016). Before recombination filtering, the ST4 and ST5 alignments contained 4,836 and 8,407 variable sites, respectively. Homologous recombination was assessed separately for each lineage using ClonalFrameML v1.12, with the lineage-specific concatenated alignment and an initial maximum-likelihood tree generated from the corresponding unfiltered alignment as inputs. Following recombination filtering, 2,295 variable sites were retained for ST4 and 3,269 variable sites were retained for ST5.

Maximum-likelihood phylogenies were reconstructed using RAxML-NG v1.2.1 (Kozlov et al., 2019). Branch support was evaluated using 100 nonparametric bootstrap replicates. Trees were treated as unrooted and visualized and annotated using iTOL v6.5.8 (Letunic and Bork, 2024). Both unfiltered and recombination-filtered phylogenetic trees were retained, with recombination-filtered trees used for the primary description of lineage-specific tree topology. Within-lineage pairwise SNP distances were calculated from the finalized recombination-filtered SNP alignments using a custom Python v3.11 workflow. Each unordered genome pair was counted once, and SNP-distance distributions were summarized using the median, interquartile range, and full range. Uncertainty in the median pairwise SNP distance was estimated by bootstrap resampling with 5,000 iterations. To evaluate the potential influence of unequal sample sizes between lineages, the ST5 dataset was additionally subsampled without replacement to 95 genomes in 5,000 iterations. No formal haplotype assignment, population-clustering algorithm, or predefined SNP-distance threshold was applied. Therefore, clusters or groupings observed in the phylogenetic trees were interpreted only as subclades or features of tree topology rather than as formally defined transmission clusters or population groups.

### 2.9 Statistical analysis

Statistical analyses were performed using IBM SPSS Statistics v27.0, GraphPad Prism v9.0, and Python v3.11. All analyses were conducted using prespecified analysis-ready datasets. Analyses not requiring isolation-source information used all 1,584 taxonomically validated *S. maltophilia* genomes, whereas source-stratified analyses were restricted to the 1,054 genomes with resolved isolation-source information, including 856 human-derived, 154 environmental, and 44 animal-derived isolates.

ARG burden and VAG burden were defined as the numbers of distinct ARGs and VAGs detected per genome, respectively. Because these variables represented discrete count data and were not assumed to follow normal distributions, they were summarized as medians and interquartile ranges (IQR). Associations between ARG and VAG burdens were assessed using two-sided Spearman rank correlation coefficients (ρ) in the complete dataset, source-resolved subset, and individual source groups. *P*-values from these five Spearman correlation tests remained unadjusted exploratory values, and the Benjamini–Hochberg multiple testing correction was not applied within this family of correlation analyses.

Source-stratified comparisons of individual ARGs and VAGs were performed using 2 × 3 contingency tables representing gene presence or absence among human, environmental, and animal isolates. Pearson’s chi-square test was used when the expected-count assumptions were satisfied. For sparse tables in which asymptotic chi-square assumptions were not adequately satisfied, an exact or fixed-margin Monte Carlo procedure with 100,000 replicates was used. The Monte Carlo procedure was also used for larger sparse contingency tables, including comparisons of major sequence-type distributions among source groups.

Genome-wide pairwise associations between individual ARGs and VAGs were evaluated across all 1,584 genomes using 2 × 2 presence/absence contingency tables. Genes lacking presence/absence variation in the analyzed dataset were excluded before statistical testing. Categorical associations were evaluated using chi-square-based tests or Fisher’s exact tests, as appropriate. Benjamini–Hochberg correction was applied separately across source-stratified ARG comparisons, source-stratified VAG comparisons, and genome-wide ARG–VAG pairwise association analyses. For the ARG–VAG pairwise analysis, correction was applied across the 1,121 valid tests. Associations with a BH-adjusted q value < 0.05 were considered statistically significant. Exact *P*-values and q values were retained in the supplementary results, whereas values < 0.001 were displayed as < 0.001 in the main tables. Effect sizes for categorical associations were quantified using Cramér’s V. For 2 × 2 contingency tables, Cramér’s V is equivalent in magnitude to the phi coefficient. Effect sizes were calculated independently of hypothesis testing using the uncorrected Pearson chi-square statistic. Cramér’s V was calculated for all 2 × 2 associations, including associations assessed using continuity-corrected chi-square or Fisher’s exact tests. All statistical tests were two-sided unless otherwise specified.

## 3 Results

### 3.1 Species-level classification of 3,343 *Stenotrophomonas* isolates based on genome-wide ANI analysis

Based on genome-wide ANI analysis, the 3,343 *Stenotrophomonas* isolates were classified into 15 species, showing a clearly uneven species distribution. *S. maltophilia* was the dominant species, accounting for nearly half of all isolates, with 1,584 strains (47.4%). The next most abundant species were *S. muris* (491 strains, 14.7%), *S. riyadhensis* (305 strains, 9.1%), *S. sepilia* (272 strains, 8.1%), and *S. geniculata* (216 strains, 6.5%). Together, these five species represented the majority of the dataset, whereas the remaining species were present at relatively low frequencies (Table 1). Several species, such as *S. nematodicola* and *S. oahuensis*, were represented by only one isolate each, indicating a long-tail distribution of rare species (Table 1).

**TABLE 1:** Species-level classification of 3,343 *Stenotrophomonas* isolates based on genome-wide average nucleotide identity analysis.

Rank	Species	Count	Percentage
1	*Stenotrophomonas maltophilia*	1,584	47.4%
2	*Stenotrophomonas muris*	491	14.7%
3	*Stenotrophomonas riyadhensis*	305	9.1%
4	*Stenotrophomonas sepilia*	272	8.1%
5	*Stenotrophomonas geniculata*	216	6.5%
6	*Stenotrophomonas pavanii*	149	4.5%
7	*Stenotrophomonas forensis*	109	3.3%
8	*Stenotrophomonas hibiscicola*	84	2.5%
9	*Stenotrophomonas indicatrix*	51	1.5%
10	*Stenotrophomonas beteli*	44	1.3%
11	*Stenotrophomonas lactitubi*	20	0.6%
12	*Stenotrophomonas cyclobalanopsidis*	8	0.2%
13	*Stenotrophomonas rhizophila*	8	0.2%
14	*Stenotrophomonas nematodicola*	1	0.0%
15	*Stenotrophomonas oahuensis*	1	0.0%

### 3.2 Temporal, geographic and host distribution of *S. maltophilia*

**FIGURE 2:**
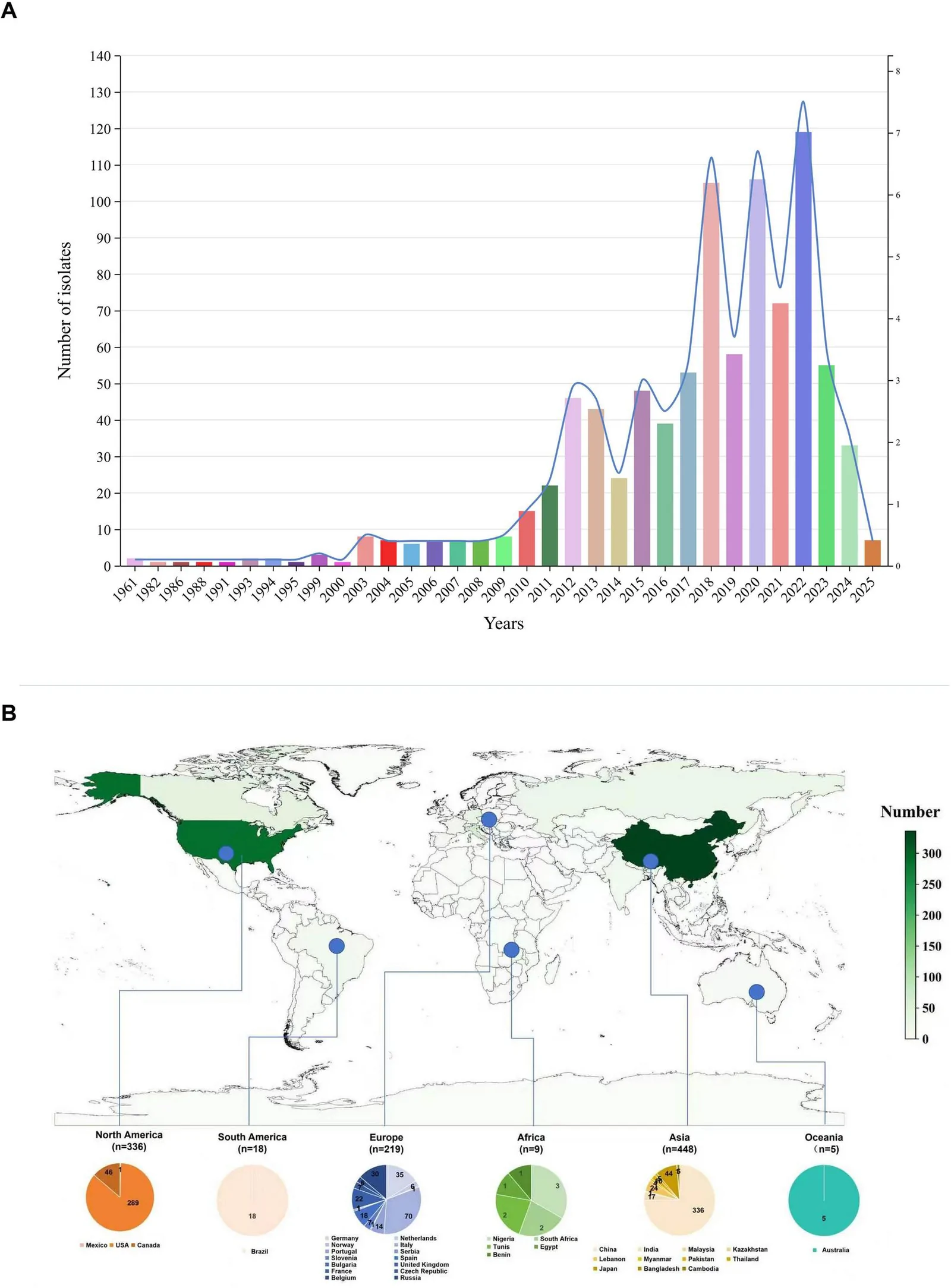
Temporal and geographic distribution of taxonomically validated *Stenotrophomonas maltophilia* genomes. **(A)** Temporal distribution of *S. maltophilia* genomes according to collection year. Collection-year information was available for 911 of the 1,584 analysis-ready genomes, whereas genomes without resolvable collection-year metadata were excluded from the year-based visualization. The number of available isolates increased markedly after 2010, with the highest number of genomes collected in 2022. **(B)** Global geographic distribution of *S. maltophilia* genomes with available country-level metadata. Among the 1,584 analysis-ready genomes, 1,044 genomes had resolvable country information and represented 35 countries across six continents. The choropleth map indicates the number of genomes contributed by each country. Pie charts summarize regional distributions and major contributing countries within each continent. Countries with fewer available genomes are grouped according to their corresponding geographic regions.

Collection-year information was available for 911 of the 1,584 genomes (57.5%), whereas 673 genomes (42.5%) lacked a resolvable collection year. Among all genomes, 119 were collected in 2022 (7.5%); this corresponded to 13.1% of the 911 genomes with resolved collection-year information. From 1957 to 2009, the number of isolates remained low, with only sporadic detections (1–8 isolates per year). A marked increase was observed after 2010, reaching a peak in 2022 (*n* = 119, 7.5%), followed by a slight decline in 2023 (Figure 2A).

Geographically, among the 1,584 genomes, 1,044 (65.9%) had resolvable country-level geographic information and represented 35 countries across six continents, whereas 540 (34.1%) had missing or unresolved geographic metadata. The majority of isolates were from Asia (*n* = 448, 28.3%), North America (*n* = 336, 21.2%), and Europe (*n* = 219, 13.8%), while fewer isolates were obtained from South America (*n* = 18, 1.1%), Africa (*n* = 18, 1.1%), and Oceania (*n* = 5, 0.3%). The top five contributing countries were China (*n* = 336, 21.2%), the United States (*n* = 289, 18.2%), Italy (*n* = 70, 4.4%), Canada (*n* = 46, 2.9%), and Japan (*n* = 44, 2.8%), representing the largest country contributors to the analyzed dataset (Figure 2B).

Among 1,054 genomes with resolved isolation-source information, 856 (81.2%) were human-derived, 154 (14.6%) were environmental, and 44 (4.2%) were animal-derived. Among the 856 human-derived genomes, the most frequently reported specimen types were sputum (276/856, 32.2%), lung tissue (109/856, 12.7%), swabs (64/856, 7.5%), blood (44/856, 5.1%), and wound samples (20/856, 2.3%). Among the 154 environmental genomes, common sources included water (46/154, 29.9%), laboratory-associated environments (18/154, 11.7%), soil (12/154, 7.8%), and vegetables (11/154, 7.1%). Among the 44 animal-derived genomes, the most frequently reported hosts were cattle (13/44, 29.5%), chickens (8/44, 18.2%), and horses (7/44, 15.9%) (Table 2).

**TABLE 2:** Hosts and sample types of *S. maltophilia* isolates worldwide.

Host (n)^a^	Sample type (n)
Homo sapiens (856)	Sputum (276), Human (180), Lung (109), Hospital (52), Swab (64), Blood (44), Wound (20), Urine (15), Other body fluids/ascites/bile (12), Feces (9), Tissue (4), Eye discharge (3), Genome (68)
Environments (154)	Environment (43), Water (46), Laboratory (18), Soil (12), Vegetable (11), Hospital (9), Metal (9), Food (1), NA (5)
Animal (44)	Horse (7), Chicken (8), Dog (2), Cattle (13), Cat (1), Amoeba (4), Duck (1), Swine (1), Insect (3), Rabbit (2), Ictalurus punctatus (1), NA (1)

^a^Amongthe 1,584 strains, only 1,054 had host and sample-type information. NA, not applicable.

### 3.3 ARG profiling and validation of the ARG identification workflow

The custom BLASTN-based screening workflow identified 12,215 ARG loci representing 67 distinct ARG designations across the 1,584 *S. maltophilia* genomes. The number of detected ARG loci per genome ranged from 3 to 25, with a mean of 7.71 loci per genome. After collapsing multiple copies of the same ARG within individual genomes, the median ARG burden was 7 distinct ARGs per genome [interquartile range (IQR), 7–8; range, 3–16]. The identified ARGs represented diverse resistance categories, including multidrug efflux-associated resistance and resistance to aminoglycosides, quinolones, β-lactams, sulfonamides, phenicols, macrolides, and tetracyclines.

**FIGURE 3:**
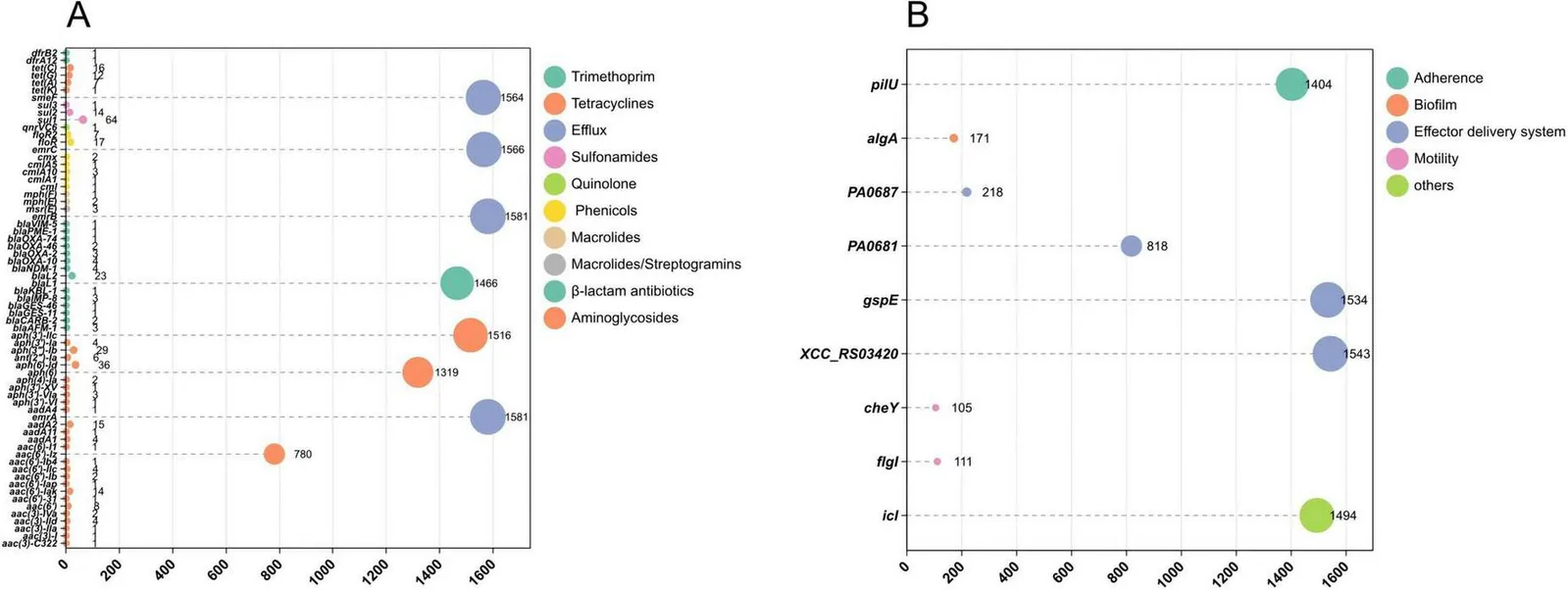
Distribution of antimicrobial resistance genes and virulence-associated genes among *Stenotrophomonas maltophilia* genomes. **(A)** Distribution and prevalence of ARGs identified across the 1,584 taxonomically validated *S. maltophilia* genomes. Each bubble represents the number of genomes carrying the corresponding ARG after collapsing multiple copies of the same gene within an individual genome. ARGs are grouped according to their associated resistance categories, including efflux, tetracycline, sulfonamide, phenicol, macrolide, macrolide/streptogramin, β-lactam, and aminoglycoside resistance. **(B)** Distribution and prevalence of virulence-associated genes identified across the same genome dataset. Each bubble represents the number of genomes containing the corresponding virulence-associated gene after collapsing multiple copies within an individual genome. Genes are grouped according to functional categories derived from VFDB annotation, including adherence, effector delivery system, immune modulation, stress survival, biofilm-associated, motility-associated, and other virulence-associated categories. The bubble size corresponds to the number of genomes in which each gene was detected.

Efflux-associated genes were among the most prevalent determinants. *emrA* and *emrB* were each detected in 1,581 of 1,584 genomes (99.8%), followed by *emrC* in 1,566 genomes (98.9%), and *smeF* in 1,564 genomes (98.7%) (Figure 3A). These prevalence values represent genome-level presence after duplicate loci of the same gene within a genome were collapsed. Intrinsic β-lactamase genes showed markedly different distributions. The metallo-β-lactamase gene *blaL1* was detected in 1,466 genomes (92.6%), whereas *blaL2* was detected in 23 genomes (1.5%). Acquired carbapenemase genes were uncommon and occurred only sporadically, including *blaNDM-1* in four genomes, *blaIMP-8* and *blaAFM-1* in three genomes each, *blaOXA-46* in two genomes, and *blaVIM-5* and *blaOXA-74* in one genome each.

To evaluate the robustness of the custom ARG identification workflow, BLASTN-derived ARG calls were independently compared with AMRFinderPlus annotations across all 1,584 *S. maltophilia* genomes. AMRFinderPlus generated 16,208 records classified as AMR, whereas records classified as STRESS were excluded from ARG concordance analysis. At the locus level, 12,214 of the 12,215 BLASTN-derived ARG calls (99.99%) showed concordant matches in the AMRFinderPlus AMR output, with only one BLASTN-derived locus not recovered by AMRFinderPlus. Conversely, AMRFinderPlus identified additional AMR-associated loci that were not detected by the nucleotide-based BLASTN workflow. Among the 16,208 AMRFinderPlus AMR loci, 3,994 loci were not recovered by the custom BLASTN workflow. These AMRFinderPlus-specific detections were predominantly identified through HMM-based searches (3,583/3,994, 89.7%), followed by BLASTP-based detection (344/3,994, 8.6%), partial protein matches (63/3,994, 1.6%), and exact protein matches (4/3,994, 0.1%). These differences reflect the distinct analytical frameworks of the two approaches, with the custom workflow relying on nucleotide-level similarity against curated reference sequences, whereas AMRFinderPlus incorporates protein-based detection, hidden Markov models, and curated AMR classification rules.

Overall, the custom BLASTN-derived ARG dataset represented a highly concordant subset of AMRFinderPlus-annotated AMR loci and provided a consistent foundation for downstream analyses of ARG burden, ARG–VAG associations, and genome-level resistance profiles.

### 3.4 Distribution of VAGs

Across the 1,584 *S. maltophilia* genomes, 7,436 VFDB-derived hits corresponding to 59 reference sequences were identified using the customized BLASTN-based screening workflow. Because multiple VFDB reference sequences may correspond to the same VAG, redundant reference entries were collapsed at the gene-symbol level before downstream analyses. After consolidation, 38 non-redundant VAGs were retained for subsequent analyses.

Based on gene-symbol-level presence/absence profiles, the mean number of VAGs was 4.7 per genome. After collapsing multiple reference hits corresponding to the same VAG gene within individual genomes, the median VAG burden was 5 distinct VAGs per genome (IQR, 4–5; range, 1–10). The identified VAGs represented diverse functional categories, including secretion systems, adhesion-associated determinants, biofilm-associated factors, motility-related components, and metabolic-associated factors.

Among the detected determinants, secretion system-associated genes were highly prevalent. The type II secretion-associated genes *XCC_RS03420* and *gspE* were detected in 1,543/1,584 genomes (97.4%) and 1,534/1,584 genomes (96.8%), respectively. The adhesion-associated gene *pilU* was detected in 1,404/1,584 genomes (88.6%), representing the most prevalent adhesion-associated VAGs (Figure 3B). Other frequently detected VAGs included *icl* (1,494/1,584, 94.3%) and *PA0681* (818/1,584, 51.6%).

### 3.5 ST distribution

After excluding 196 genomes with undetermined STs, 1,388 isolates were assigned to 234 distinct STs. Among these typeable isolates, the most prevalent sequence types were ST5 (*n* = 113, 8.1%), ST4 (*n* = 95, 6.8%), ST31 (*n* = 68, 4.9%), ST162 (*n* = 67, 4.8%), and ST115 (*n* = 45, 3.2%).

Geographic variation was observed in the frequencies of the major STs within the sampled dataset. ST31, ST4, and ST115 were frequently represented among genomes from China, whereas ST208 was frequently represented among genomes from Japan. In the United States, commonly observed types included ST5, ST39, and ST26, while ST199 was frequently represented among genomes from Canada. These patterns describe the composition of the available public-genome dataset and should not be interpreted as estimates of country-level prevalence because sequencing and database submission were uneven across regions.

### 3.6 Source-associated differences in ARG and VAG profiles

Source-stratified analyses were performed using genomes with confidently resolved isolation-source metadata. At the resistance-category level, efflux-, aminoglycoside-, β- lactam-, and quinolone-associated determinants were broadly distributed across all three source groups, and no clear source-associated differences were observed.

**FIGURE 4:**
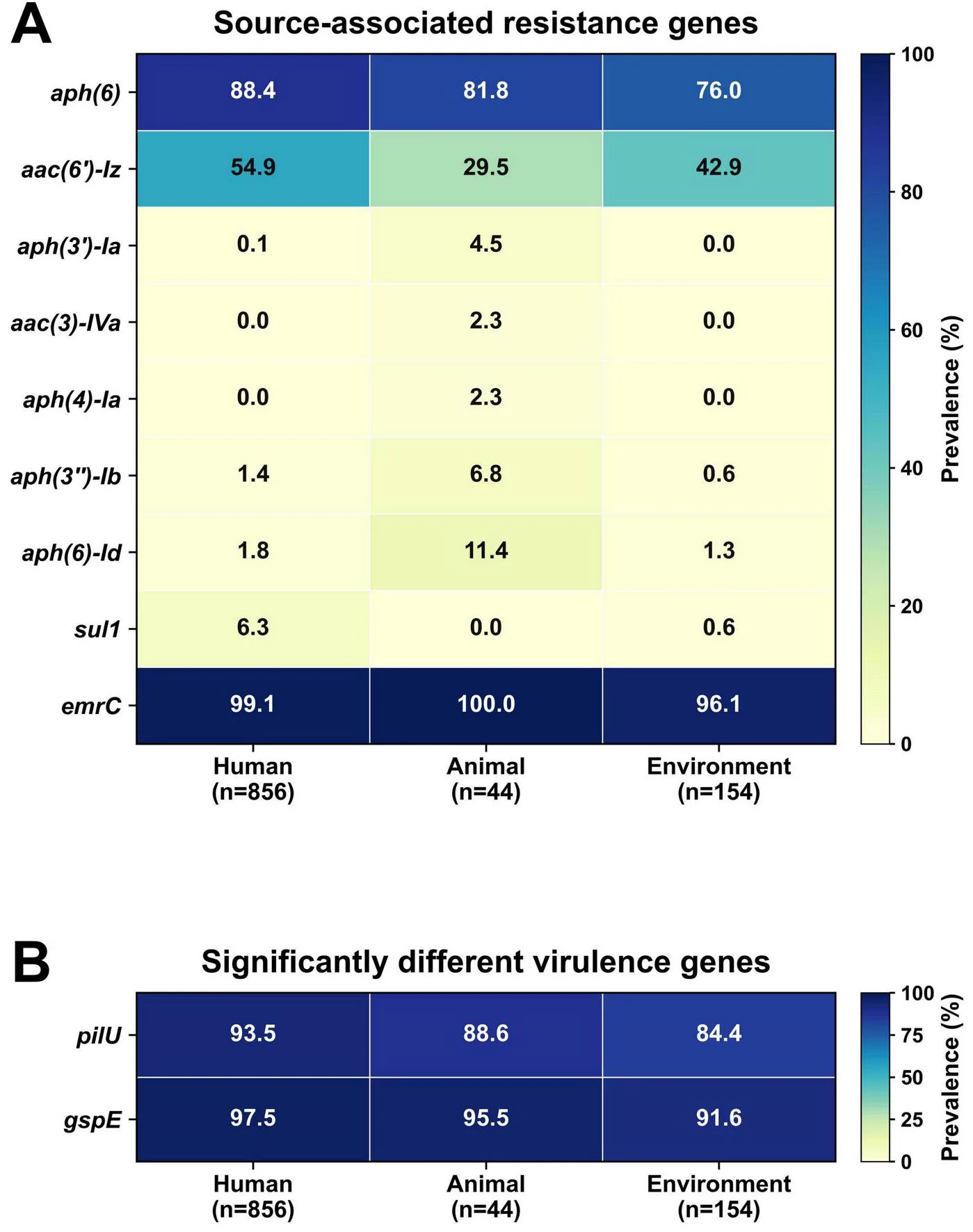
Source-associated differences in antimicrobial resistance and virulence-associated gene profiles among *Stenotrophomonas maltophilia* genomes. **(A)** Heatmap showing the prevalence of selected ARGs across *S. maltophilia* genomes with resolved isolation-source information, including 856 human-derived, 154 environmental, and 44 animal-derived genomes. Values represent the percentage of genomes containing each ARG within the corresponding source category. ARGs displayed were selected based on source-associated comparisons of individual ARG prevalence. **(B)** Heatmap showing virulence-associated genes with significant source-associated differences after Benjamini–Hochberg correction. Values represent the percentage of genomes containing each virulence-associated gene within each source category. Only genes that remained statistically significant after multiple-testing correction are displayed. Source-associated comparisons were performed using categorical association tests followed by Benjamini–Hochberg correction. The color intensity represents gene prevalence (%) within each source category.

Among the 67 ARGs identified, genes showing presence/absence variation were evaluated using 2 × 3 contingency-table analyses, followed by Benjamini–Hochberg correction. Five ARGs differed significantly among the three source groups at a BH-adjusted *q*0.05: the aminoglycoside-resistance genes *aac(6*’*)-Iz, aph(3*”*)-Ib, aph(6)*, and *aph(6)-Id*, and the sulfonamide-resistance gene *sul1*. The *aph(6)* gene was prevalent across all three source groups, whereas *aph(3*”*)-Ib* and *aph(6)-Id* showed relatively higher prevalence among animal-derived isolates (Figure 4A; Supplementary Table S8).

Among the 38 VAGs examined, two genes, *pilU* and *gspE*, showed significant source-associated differences after Benjamini–Hochberg correction. The prevalence of *pilU* was 93.5% (800/856) in human-derived isolates, 84.4% (130/154) in environmental isolates, and 88.6% (39/44) in animal-derived isolates. The corresponding prevalence of *gspE* was 97.5% (835/856), 91.6% (141/154), and 95.5% (42/44), respectively. In contrast, *algA*, *PA0687*, and *flgI* showed no significant source-associated differences after multiple-testing correction (Figure 4B; Supplementary Table S9).

This study adopted the 2 × 2 presence/absence contingency table approach to explore the pairwise co-occurrence association characteristics between antibiotic resistance genes and virulence factors across the whole-genome dataset. After Benjamini-Hochberg multiple testing correction, multiple ARG-VF combinations exhibited significant associations (adjusted *q* < 0.001). Among these pairs, *blaL1* showed the strongest correlation with *cheY* (Cramér’s *V* = 0.775), followed by *blaL1* and *gspE* (Cramér’s *V* = 0.554), as well as *aph(6)* and *pilU* (Cramér’s *V* = 0.532). In addition, *blaL2* was significantly associated with *XCC_RS03420* (Cramér’s *V* = 0.745), yet these two genes rarely co-occurred. The results indicate that *blaL1* and *aph(6)* may undergo co-selection alongside virulence genes involved in motility and secretion systems (Table 3A).

**TABLE 3A:** Results of pairwise association tests for ARG-VF pairs (top 10 sorted by BH *q*-value).

ARG	VF	2 × 2 count ++/+−/−+/−	Minimum expected count	*P*-value	BH *q*-value	Cramér’s V
*blaL1*	*cheY*	17/1,449/88/30	7.822	< 0.001	<0.001	0.775^a^
*blaL1*	*gspE*	1,460/6/74/44	3.725	< 0.001	<0.001	0.554^a^
*aph(6)*	*pilU*	1,269/50/135/130	30.114	< 0.001	<0.001	0.532^a^
*aph(6)*	*cheY*	10/1,309/95/170	17.566	< 0.001	<0.001	0.526^a^
*blaL2*	*icl*	0/23/1,494/67	1.307	< 0.001	<0.001	0.495^b^
*blaL2*	*cheY*	23/0/82/1,479	1.525	< 0.001	<0.001	0.456^b^
*blaL1*	*XCC_RS03420*	1,457/9/86/32	3.054	< 0.001	<0.001	0.438^a^
*aph(6)*	*gspE*	1313/6/221/44	8.365	< 0.001	<0.001	0.345^a^
*blaL2*	*XCC_RS03420*	0/23/1,543/18	0.595	< 0.001	<0.001	0.745^b^
*aph(3)-IIc*	*icl*	1454/62/40/28	3.864	< 0.001	<0.001	0.325^a^

ARG, antimicrobial resistance gene; VF, virulence factor; 2 × 2 count ++/+−/−+/−, number of genomes positive for both ARG and VF/ARG-positive VF-negative/ARG-negative VF-positive/negative for both; BH *q*-value, Benjamini-Hochberg adjusted *P*-value; Cramér’s V, effect size for categorical association.

^a^Pearson χ^2^ test;

^b^Fisher’s exact test.

Within the source-resolved subset (*n* = 1,054), the median ARG burden was 8 genes per genome (IQR, 7–8), and the median VAG burden was 5 genes per genome (IQR, 4–5). ARG and VAG burdens showed a statistically significant but very weak positive correlation across source-resolved genomes (Spearman’s ρ = 0.061, *P* = 0.048). In source-specific analyses, a weak positive correlation was observed among environmental isolates (ρ = 0.245, *P* = 0.002), whereas no significant correlations were detected among human-derived (ρ = 0.031, *P* = 0.367) or animal-derived isolates (ρ = 0.002, *P* = 0.990) (Table 3B). These findings suggest that source-associated differences were limited to specific ARGs and VAGs rather than reflecting broad differences in overall ARG or VAG burden.

**TABLE 3B:** Spearman correlation analysis between ARG burden and VF burden.

Data set	*N*	Rho	*P*-value	ARG median (IQR)	VF median (IQR)
All genomes	1,584	0.114	< 0.001	7 (7–8)	5 (4–5)
Known-source genomes	1,054	0.061	0.048	8 (7–8)	5 (4–5)
Human	856	0.031	0.367	8 (7–8)	5 (4–5)
Environment	154	0.245	0.002	7 (7–8)	5 (4–5)
Animal	44	0.002	0.990	7 (7–7)	5 (4–5)

ARG, antimicrobial resistance gene; VF, virulence factor; N, number of genomes; Rho, Spearman’s rank correlation coefficient; IQR, interquartile range. These five Spearman correlation analyses report uncorrected *P*-values for exploratory interpretation; the Benjamini–Hochberg correction was applied to comparisons between ARG/VF sources and pairwise association tests between ARGs and VFs, but was not applied to these five Spearman correlation analyses.

### 3.7 Distribution differences of ARGs and VAGs among major STs

**FIGURE 5:**
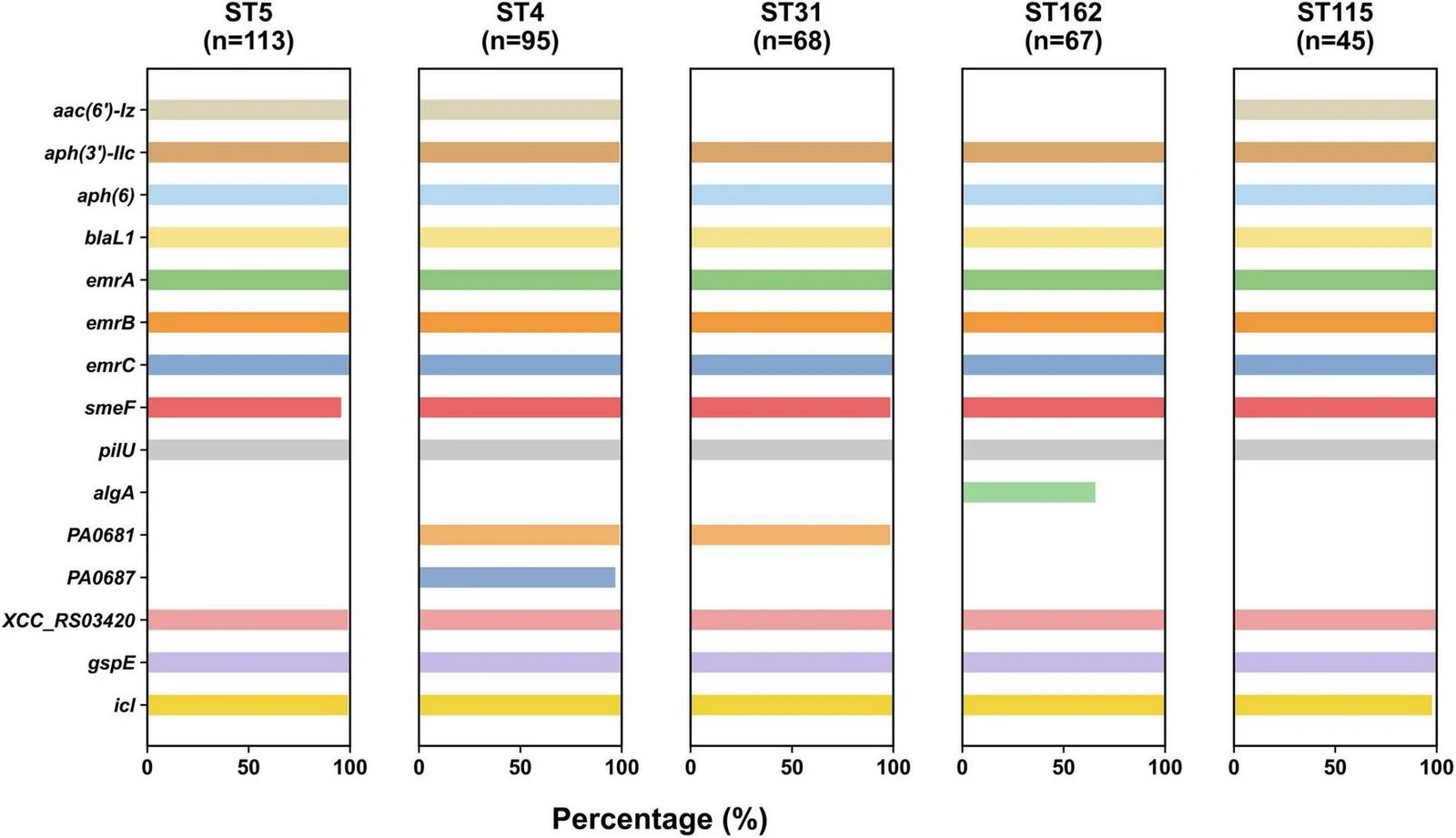
Sequence-type-associated distribution of antimicrobial resistance and virulence-associated genes in *Stenotrophomonas maltophilia*. The prevalence of selected ARGs and virulence-associated genes among the five major STs identified in the analyzed *S. maltophilia* genome dataset is shown. The included STs were ST5 (*n* = 113), ST4 (*n* = 95), ST31 (*n* = 68), ST162 (*n* = 67), and ST115 (*n* = 45). Values represent the percentage of genomes within each ST containing the corresponding gene. The displayed genes include representative intrinsic resistance determinants, acquired resistance-associated genes, and prevalent virulence-associated genes selected based on their distribution patterns among major STs.

The distribution of major ARGs and VAGs was compared among five predominant STs, including ST5, ST4, ST31, ST162, and ST115. Overall, several ARGs were highly conserved across these major STs. The efflux-associated genes *emrA*, *emrB*, *emrC* and *smeF*, the β-lactam resistance gene *blaL1*, the aminoglycoside resistance genes *aph(3*’*)-IIc* and *aph(6)* were widely distributed in nearly all isolates of the five STs. In contrast, several genes showed ST-specific distribution patterns. The aminoglycoside resistance gene *aac(6*’*)-Iz* was highly prevalent in ST5, ST4, and ST115, but was absent or much less frequent in ST31 and ST162. Among VAGs, *pilU*, *XCC_RS03420*, *gspE*, and *icl* were broadly distributed across the major STs, suggesting that adherence-, effector delivery system-, and metabolism-related virulence traits are relatively conserved. However, *algA* was mainly detected in ST162, whereas *PA0681* was more frequently observed in ST4 and ST31. PA0687 was detected mainly in ST4 but was absent from the other major STs (Figure 5).

### 3.8 Distribution of predicted MGEs and predicted ARG–MGE associations

Across the 1,584 *S. maltophilia* genomes, 38,194 non-redundant predicted MGE regions were identified, with a combined sequence length of 868.8 Mb. ISs were the most frequently predicted MGE category, comprising 17,756 regions and accounting for 46.5% of all non-redundant predicted MGE regions. Phage- or prophage-associated regions were predicted in 1,583 genomes (99.9%), whereas plasmid-associated sequences were predicted in 1,471 genomes (92.9%) using the broad union criterion, under which a contig classified as plasmid-associated by either geNomad or MOB-suite was retained. Predicted plasmid-associated and phage/prophage-associated sequences accounted for 62.1 and 25.9% of the total predicted MGE sequence length, respectively. ICE- or IME-associated regions were identified in 801 genomes (50.6%). Predicted transposons, integrons, and other MGE categories were less frequent and were identified in 277 (17.5%), 233 (14.7%), and 34 (2.1%) genomes, respectively.

Predicted ARG–MGE associations varied among MGE categories. Among the 1,471 genomes containing predicted plasmid-associated sequences, 586 (39.8%) contained at least one ARG located on a contig classified as plasmid-associated. Predicted same-contig coordinate overlaps between ARGs and ICE/IME, integron, insertion-sequence, and phage/prophage regions were detected in 72 of 801 (9.0%), 35 of 233 (15.0%), 18 of 1,584 (1.1%), and 11 of 1,583 (0.7%) genomes containing the corresponding predicted MGE categories, respectively. No ARGs overlapped predicted transposon regions under the predefined same-contig and coordinate-overlap criteria.

**FIGURE 6:**
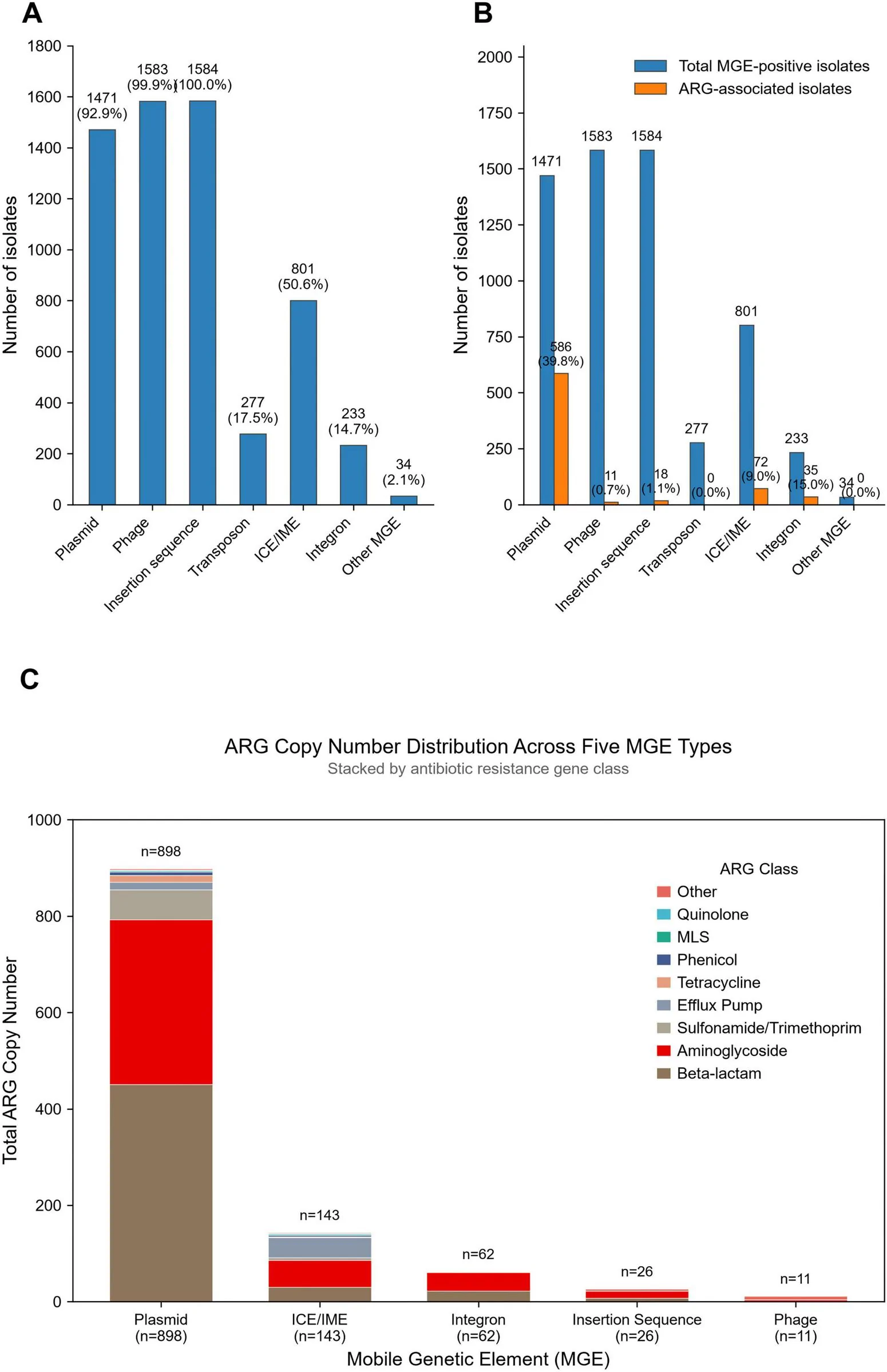
Distribution of mobile genetic elements and predicted ARG–MGE associations in *Stenotrophomonas maltophilia* genomes. **(A)** Distribution of predicted MGEs identified among the 1,584 taxonomically validated *S. maltophilia* genomes. Bars represent the number and proportion of genomes containing each MGE category, including plasmid-associated sequences, phage/prophage elements, insertion sequences, integrative conjugative elements/integrative mobilizable elements (ICEs/IMEs), integrons, and other MGEs. **(B)** Distribution of ARG-associated MGEs among genomes containing the corresponding MGE categories. Blue bars indicate the total number of genomes with each predicted MGE category, whereas orange bars indicate genomes in which ARGs were predicted to be associated with the corresponding MGE category based on sequence-level co-localization criteria. ARG–MGE associations were defined based on ARG localization on the same contig as the predicted MGE, with coordinate overlap required for defined MGE regions. **(C)** Distribution of ARG copy numbers associated with different MGE categories. Stacked bars show the total number of ARG copies detected within each predicted MGE category, stratified by antimicrobial resistance class. ARG–MGE relationships represent computational predictions of sequence-level co-localization and do not demonstrate confirmed plasmid localization, intact mobile element structure, mobilization potential, or horizontal gene transfer.

In total, 1,140 predicted ARG–MGE co-localization events were identified across the five ARG-associated MGE categories. Predicted plasmid-associated sequences represented the largest predicted category, accounting for 898 events (78.8%), followed by ICEs/IMEs (143, 12.5%), integrons (62, 5.4%), ISs (26, 2.3%), and phage/prophage-associated regions (11, 1.0%). The ARGs most frequently detected on predicted plasmid-associated contigs were blaL1 (*n* = 422) and *aph(3*’*)-IIc* (*n* = 202). The 898 plasmid-associated events represent a broad prediction set supported by at least one of the two plasmid-classification tools and should not all be interpreted as high-confidence plasmid assignments. Concordant and single-tool predictions, together with genome identifiers, contig coordinates, assembly-contiguity categories, and tool-specific evidence, are reported in the contig-level evidence table (Figure 6; Supplementary Table S10).

Of the 1,584 genomes, 69 were represented by a single contig and were classified as the higher-contiguity subset, whereas the remaining 1,515 were multi-contig draft assemblies. Single-contig status was not considered proof of complete genome closure or long-read sequencing. Consequently, all reported ARG–MGE relationships represent computationally predicted sequence-level co-localization and do not establish confirmed plasmid carriage, intact mobile-element localization, mobilizability, or horizontal transferability.

### 3.9 Pangenome comparison between ST4 and ST5 *S. maltophilia* strains

Separate pangenome analyses of the ST4 and ST5 genomes identified differences in orthogroup-occupancy and gene-assignment metrics. Among the 95 ST4 genomes, 7,220 orthogroups were identified. Of these, 3,597 were strict-core orthogroups present in all 95 genomes (49.8%). No separate near-core category was present because an orthogroup detected in 94 of 95 genomes had an occupancy of 98.95% and therefore did not meet the ≥ 99% threshold. A total of 1,074 orthogroups were classified as shell orthogroups, occurring in 15–94 genomes (14.9%), and 2,301 were classified as cloud orthogroups, occurring in 2–14 genomes (31.9%). An additional 248 orthogroups were strain-specific and occurred in only one genome (3.4%). These mutually exclusive categories summed to the total of 7,220 orthogroups: 3,597 strict-core, 0 near-core, 1,074 shell, 2,301 cloud, and 248 strain-specific orthogroups. Overall, 421,540 of 433,440 predicted genes (97.3%) were assigned to orthogroups, whereas 11,900 genes (2.7%) remained unassigned. The ST4 occupancy distribution used for these classifications is reported in the OrthoFinder summary output.

Among the 113 ST5 genomes, 7,043 orthogroups were identified. Of these, 3,233 were strict-core orthogroups present in all 113 genomes (45.9%), and 460 were near-core orthogroups present in 112 of 113 genomes (6.5%). Together, these categories yielded 3,693 orthogroups meeting the ≥ 99% core threshold (52.4%). A further 1,146 orthogroups were classified as shell orthogroups, occurring in 17–111 genomes (16.3%), and 2,111 were classified as cloud orthogroups, occurring in 2–16 genomes (30.0%). Ninety-three orthogroups were strain-specific and occurred in only one genome (1.3%). These mutually exclusive categories also summed to the total of 7,043 orthogroups: 3,233 strict-core, 460 near-core, 1,146 shell, 2,111 cloud, and 93 strain-specific orthogroups. Of the 516,311 predicted genes, 512,245 (99.2%) were assigned to orthogroups, whereas 4,066 genes (0.8%) remained unassigned.

Comparison of the mutually exclusive occupancy categories showed that ST4 had higher proportions of strict-core orthogroups (49.8% versus 45.9%), cloud orthogroups (31.9% versus 30.0%), and strain-specific orthogroups (3.4% versus 1.3%). ST5 had a distinct near-core component (6.5% versus 0%) and a slightly higher proportion of shell orthogroups (16.3% versus 14.9%). When shell, cloud, and strain-specific orthogroups were collectively defined as the accessory genome, the accessory proportions were 50.2% in ST4 and 47.6% in ST5. However, when only orthogroups present in more than one genome but below the ≥ 99% core threshold were considered, the combined shell-plus-cloud proportions were similar between ST4 and ST5 (46.7% versus 46.2%). ST4 also had a higher proportion of unassigned genes, whereas ST5 had a higher orthogroup-assignment rate.

**FIGURE 7:**
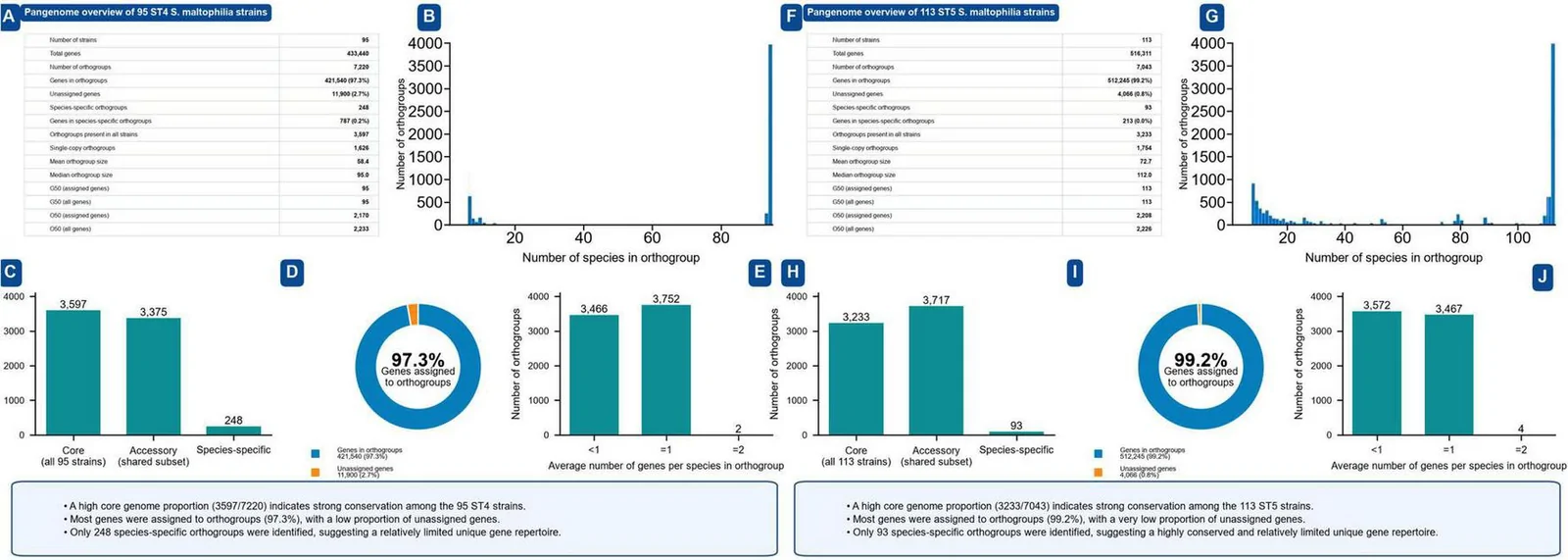
Comparative pangenome characteristics of ST4 and ST5 *Stenotrophomonas maltophilia* lineages. Pangenome analysis was performed separately for 95 ST4 and 113 ST5 *S. maltophilia* genomes using OrthoFinder v2.5.5. **(A–E)** Pangenome characteristics of the ST4 lineage. **(F–J)** Pangenome characteristics of the ST5 lineage. **(A,F)** Summary statistics of lineage-specific pangenome composition, including the number of genomes analyzed, total predicted genes, orthogroup assignment, unassigned genes, total orthogroups, and orthogroup-related metrics. **(B,G)** Distribution of orthogroup occupancy across genomes within each lineage. The x-axis represents the number of genomes containing each orthogroup, and the y-axis represents the number of orthogroups observed at each occupancy level. **(C,H)** Distribution of strict-core, accessory, and strain-specific orthogroups. Strict-core orthogroups were defined as orthogroups present in 100% of genomes within the corresponding lineage. Accessory orthogroups comprised all non-core orthogroups, including shell, cloud, and strain-specific orthogroups according to predefined genome-occupancy thresholds. **(D,I)** Proportion of predicted genes assigned to orthogroups versus unassigned genes within each lineage. **(E,J)** Distribution of average gene copy number per genome within orthogroups. Orthogroup categories were defined independently for each lineage based on genome occupancy thresholds. These analyses describe the observed pangenome composition of the sampled ST4 and ST5 datasets and were not used to infer open or closed pangenome status.

Although ST4 contained slightly more observed orthogroups than ST5, the raw cumulative counts were considered sensitive to the unequal lineage sample sizes and were not interpreted independently as evidence of greater pangenome diversity. No pangenome-accumulation model or formal openness analysis was performed; therefore, neither lineage was classified as having an open or closed pangenome. The results instead describe metric-specific differences in orthogroup occupancy, rare-gene content, and gene-assignment rates between the sampled ST4 and ST5 genomes (Table 4; Figure 7).

**TABLE 4:** Metric-specific quantitative comparison of pangenome composition and core-genome sequence variation between ST4 and ST5 *S. maltophilia.*

Analysis domain	Metric	ST4	ST5	Interpretation/uncertainty
Sampling	Genomes included	95	113	Unequal sample sizes were considered in cumulative-count comparisons
Gene assignment	Total predicted genes	433,440	516,311	Cumulative and sample-size-sensitive
Gene assignment	Genes assigned to orthogroups	421,540	512,245	–
Gene assignment	Orthogroup-assignment rate	97.3%	99.2%	Higher in ST5
Gene assignment	Unassigned genes	11,900 (2.7%)	4,066 (0.8%)	Higher proportion in ST4
Pangenome	Total observed orthogroups	7,220	7,043	Not interpreted alone as lineage-wide diversity
Pangenome	Strict-core, 100% prevalence	3,597 (49.8%)	3,233 (45.9%)	Higher strict-core proportion in ST4
Pangenome	Near-core, ≥ 99% but < 100%	0 (0.0%)	460 (6.5%)	ST5 near-core orthogroups occurred in 112/113 genomes
Pangenome	Total ≥ 99% core	3,597 (49.8%)	3,693 (52.4%)	Higher ≥ 99%-core proportion in ST5
Pangenome	Shell	1,074 (14.9%)	1,146 (16.3%)	ST4: 15–94 genomes; ST5: 17–111 genomes
Pangenome	Cloud	2,301 (31.9%)	2,111 (30.0%)	ST4: 2–14 genomes; ST5: 2–16 genomes
Pangenome	Strain-specific	248 (3.4%)	93 (1.3%)	Higher rare-gene component in ST4
Pangenome	Accessory, shell + cloud + strain-specific	3,623 (50.2%)	3,350 (47.6%)	Derived aggregate; not added again when checking category sums
Core alignment	Concatenated alignment length	1,379,917 bp	1,853,398 bp	Lineage-specific alignment lengths
Core SNPs	Variable sites before recombination filtering	4,836	8,407	Raw counts depend partly on alignment length and sample size
Core SNPs	Pre-filter variable-site density	3.50 SNPs/kb	4.54 SNPs/kb	29.4% higher in ST5
Recombination filtering	Variable sites retained after ClonalFrameML	2,295	3,269	ST5 retained 974 more sites
Recombination filtering	Sites excluded	2,541 (52.5%)	5,138 (61.1%)	Predicted recombinant regions accounted for a larger fraction in ST5
Pairwise SNP distance	Number of unique genome pairs	4,465	6,328	[n(n-1)/2] comparisons
Pairwise SNP distance	Median [IQR]	108 [90–126]	127 [67–156]	Exact alignment source must be stated
Pairwise SNP distance	Range	0–367	0–576	–
Pairwise SNP distance	Genome-bootstrap 95% CI for median	102–114	111–140	Genome-level bootstrap, 5,000 iterations
Pairwise SNP distance	Median pairwise SNP distance	108 SNPs	127 SNPs	19 SNPs (bootstrap 95% CI, 2–33)
Sample-size control	Median after ST5 subsampling to 95 genomes	–	128	95% subsampling interval, 121–133; 5,000 iterations

“−” indicates that the metric is not applicable to the corresponding column; it does not represent zero or missing data. 1. Orthogroup categories were mutually exclusive. Strict-core, near-core, shell, cloud, and strain-specific counts summed to the total number of observed orthogroups. 2. The accessory category was a derived aggregate comprising shell, cloud, and strain-specific orthogroups and was not included again when calculating the total. 3. Because 94/95 corresponds to 98.95%, no ST4 orthogroup met the ≥ 99% but < 100% near-core definition. In ST5, 112/113 corresponds to 99.12%. 4. Pangenome counts and proportions were descriptive summaries of the sampled genomes. No pangenome-accumulation or openness model was fitted, and neither lineage was classified as having an open or closed pangenome.5. Pairwise SNP distances are non-independent. Confidence intervals were therefore estimated by resampling genomes rather than individual genome pairs.

### 3.10 Phylogenetic relationships of the dominant ST4 and ST5 lineages

Lineage-specific core-genome phylogenies were generated for the 95 ST4 and 113 ST5 genomes. Before homologous-recombination filtering, the concatenated core-gene alignments contained 4,836 variable sites in ST4 and 8,407 in ST5. Following recombination filtering, 2,295 variable sites were retained in ST4 and 3,269 in ST5, corresponding to the exclusion of 52.5 and 61.1% of the initial variable sites, respectively. Predicted recombinant regions therefore accounted for a larger proportion of the unfiltered variable sites in ST5.

**FIGURE 8:**
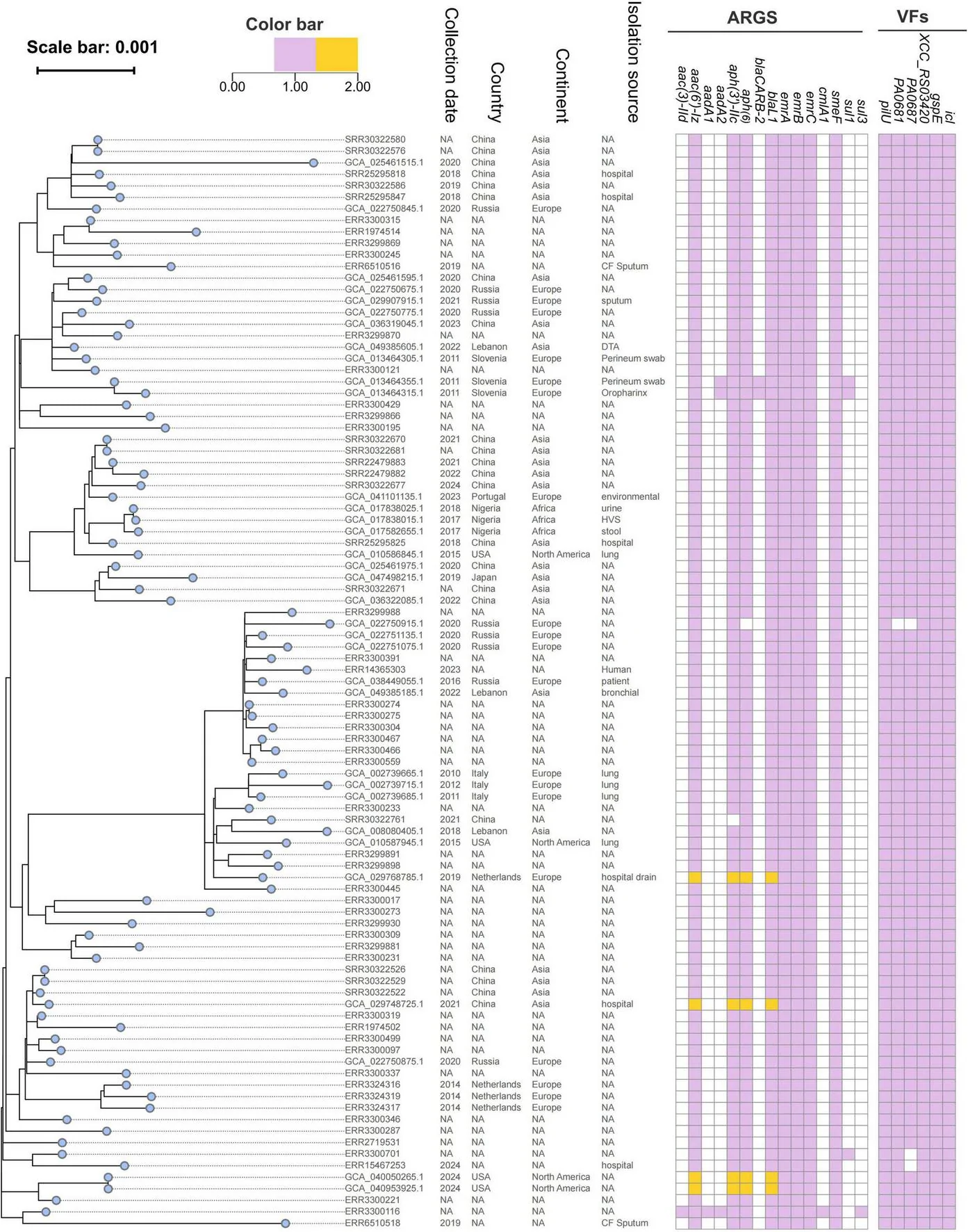
Integrated phylogenomic and genomic characterization of ST4 *Stenotrophomonas maltophilia* genomes. A maximum-likelihood phylogenetic tree was reconstructed based on concatenated single-copy core-gene alignments after homologous recombination filtering using ClonalFrameML v1.12. Genome metadata and genomic characteristics were integrated with the phylogenetic framework. The phylogenetic tree displays the genetic relationships among ST4 genomes, with branch lengths representing nucleotide substitutions per site. The annotation tracks summarize available metadata, including collection year, country of isolation, continent, and isolation source. The right-side heatmaps show the genome-level distribution of selected antimicrobial resistance genes (ARGs) and virulence-associated genes. ARG and virulence-associated gene profiles were defined based on gene presence or absence after collapsing duplicate loci within individual genomes. Missing metadata are indicated as NA. The scale bar represents nucleotide substitutions per site.

**FIGURE 9:**
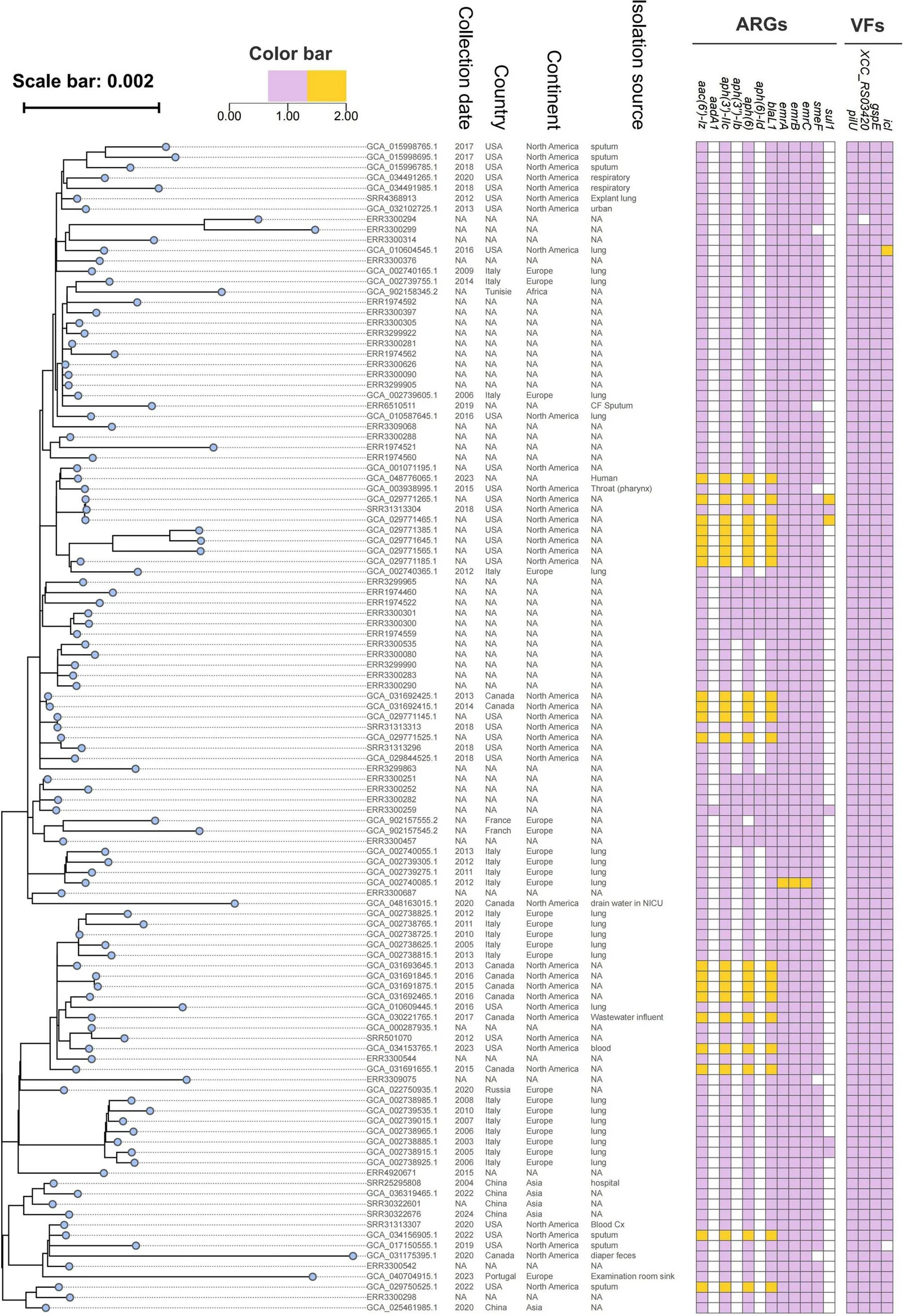
Integrated phylogenomic and genomic characterization of ST5 *Stenotrophomonas maltophilia* genomes. A maximum-likelihood phylogenetic tree was reconstructed based on concatenated single-copy core-gene alignments after homologous recombination filtering using ClonalFrameML v1.12. Genome metadata and genomic characteristics were integrated with the phylogenetic framework. The phylogenetic tree displays the genetic relationships among ST5 genomes, with branch lengths representing nucleotide substitutions per site. The annotation tracks summarize available metadata, including collection year, country of isolation, continent, and isolation source. The right-side heatmaps show the genome-level distribution of selected ARGs and virulence-associated genes. ARG and virulence-associated gene profiles were defined based on gene presence or absence after collapsing duplicate loci within individual genomes. Missing metadata are indicated as NA. The scale bar represents nucleotide substitutions per site.

Both phylogenies contained multiple subclades, with isolates from different countries, continents, collection years, and isolation sources interspersed throughout the trees (Figures 8, 9). No strict separation by geographic origin or isolation source was evident from the tree topology. Although some closely related groups contained isolates from the same country or similar specimen types, no formal clustering algorithm or predefined SNP-distance threshold was applied; these groupings were therefore described as subclades rather than as defined populations, transmission clusters, or outbreak groups. Pairwise SNP-distance analysis showed a median distance of 108 SNPs in ST4 (IQR, 90–126; range, 0–367; 4,465 genome pairs) and 127 SNPs in ST5 (IQR, 67–156; range, 0–576; 6,328 genome pairs). Genome-level bootstrap 95% intervals were 102–114 SNPs for ST4 and 111–140 SNPs for ST5. After ST5 was repeatedly subsampled to 95 genomes, its median pairwise distance remained 128 SNPs, with a 95% subsampling interval of 121–133 SNPs. These results indicate greater core-genome sequence separation in ST5 according to the retained variable-site count and pairwise SNP-distance metrics.

## 4 Discussion

In this study, we performed a large-scale genomic analysis of publicly available *Stenotrophomonas* isolates and demonstrated substantial species-level heterogeneity within the genus. Genome-wide ANI-based taxonomic verification classified 3,343 high-quality genomes into 15 *Stenotrophomonas* species, with *S. maltophilia* accounting for nearly half of the collection. These findings highlights both the predominance of *S. maltophilia* among publicly available *Stenotrophomonas* genomes and the taxonomic complexity of datasets initially assigned to this genus (Yu and Wang, 2024). The long-tail distribution of low-abundance species further suggests that rare or undersampled *Stenotrophomonas* species may remain underrepresented in routine genomic surveillance and public databases (Vinuesa et al., 2018; Zhao et al., 2024).

The temporal distribution of the *S. maltophilia* genomes showed an apparent increase in publicly available isolates after 2010, with the largest number deposited for isolates collected in 2022. This pattern likely reflects the expanding use of WGS and increased deposition of bacterial genome data rather than a direct increase in the incidence or disease burden of *S. maltophilia*. Isolates with available geographic information originated from 35 countries, supporting the broad global distribution of this species. Nevertheless, most genomes were contributed by countries in Asia, North America, and Europe, indicating substantial geographic sampling bias. Human-derived isolates, particularly those obtained from respiratory and other clinical specimens, were also overrepresented, consistent with the clinical importance of *S. maltophilia* as an opportunistic pathogen (Mojica et al., 2022). The presence of environmental and animal-derived isolates nevertheless reinforces the view that *S. maltophilia* occupies multiple ecological reservoirs and is not restricted to clinical settings (Gröschel et al., 2020; Brooke, 2021).

The resistome analysis revealed a broad repertoire of ARGs, although the most frequently detected genes were primarily associated with intrinsic resistance. Efflux-associated genes were widely distributed across the dataset, indicating that multidrug efflux systems are common components of the *S. maltophilia* resistance background (Zhang et al., 2001). Similarly, the distribution of intrinsic β-lactamase was consistent with the characteristic β-lactam resistance of this species (Sapula et al., 2025). In contrast, acquired carbapenemase genes were uncommon. Although rare, such genes remain epidemiologically relevant because their presence may further restrict therapeutic options and may indicate horizontal acquisition of clinically important resistance determinants (Li et al., 2023; Bostanghadiri et al., 2024).

VAG-gene analysis showed that several genes associated with secretion, motility, and adhesion were widely distributed among the analyzed genomes. The frequent detection of *XCC_RS03420*, *gspE*, and *pilU* suggests that these genes are common components of the genomic background represented in the present dataset. Adhesion- and secretion-associated factors may contribute to the capacity of *S. maltophilia* to colonize both host-associated and environmental surfaces (Wang et al., 2024). Compared with ARG profiles, VAG presence/absence patterns showed less variation among the major ST lineages examined, suggesting relative stability of the detected VAG repertoire within these lineages rather than uniform conservation of the entire virulome across the species (Drebes Dörr et al., 2026). Moreover, gene detection alone does not establish expression or functional contribution to virulence, which would require transcriptomic, phenotypic, or experimental validation.

Source-stratified analyses based on the reconciled cohort of 1,054 source-resolved genomes provided additional insight into the ecological distribution of resistance and virulence determinants. Most high-prevalence resistance categories were broadly distributed among human-, environmental-, and animal-derived isolates, whereas only selected ARGs and VAGs showed significant differences among the three source groups after Benjamini–Hochberg correction. This pattern suggests that source-associated variation was concentrated in particular genes rather than reflecting broad differences in the overall resistance or virulence repertoires. Such differences may be influenced by antimicrobial exposure, ecological selection, host association, geographic structure, or differences in lineage composition. They should not, however, be interpreted as direct evidence of source-specific adaptation or causation. The source groups were markedly imbalanced, particularly because the animal-derived subset was comparatively small, and residual confounding by geography, sampling period, healthcare exposure, and population structure could not be excluded (Mercier-Darty et al., 2020; Klimkaitė et al., 2025).

The ARG–VAG burden analyses further indicated that ARGs and VAGs counts were not strongly coupled at the genome level. Although a statistically significant positive correlation was detected across the complete dataset, its magnitude was weak. The association was further attenuated in the source-resolved subset and was detectable in environmental isolates but not in the human- or animal-derived groups. These findings indicate that genomes carrying a larger number of detected ARGs did not necessarily carry a proportionally larger number of VAGs. Pairwise presence/absence analyses nevertheless identified significant associations between selected ARGs and VAGs after correction for multiple testing, with some pairs showing moderate or strong effect sizes. Such associations may arise from shared lineage backgrounds, physical linkage, correlated gene retention, co-selection, or exposure to common ecological pressures. Statistical co-occurrence does not demonstrate direct functional interaction between resistance and virulence determinants. Analyses that explicitly account for phylogenetic population structure, together with examination of genomic context and experimental validation, will be required to distinguish clonal or linkage effects from biologically functional relationships.

MLST analysis revealed extensive ST heterogeneity among global *S. maltophilia* genomes. ST5 and ST4 were the most frequently represented lineages in the current dataset, consistent with previous reports that these STs are recurrently detected among clinical or healthcare-associated isolates, although the dominant STs vary among geographic regions and study populations (Kaiser et al., 2009; Gröschel et al., 2020). Their predominance in the present collection should therefore be interpreted as a feature of the publicly available dataset rather than as a direct estimate of their global prevalence. *S. maltophilia* has been implicated in healthcare-associated transmission and nosocomial outbreaks (Guyot et al., 2013; Cristina et al., 2024; Kazak et al., 2024; Khan et al., 2026). In the present study, some ST4 and ST5 isolates from the same country or from similar clinical or hospital-associated sources occurred within closely related phylogenetic subclades. However, the available metadata generally lacked hospital-level epidemiological information, including ward location, patient linkage, precise sampling site, and sufficiently detailed collection dates. These phylogenetic patterns are compatible with close genetic relatedness and possible local persistence or dissemination, but they may also reflect uneven sampling or the broader distribution of successful clonal lineages. Therefore, the observed subclades should not be interpreted as confirmed transmission clusters or outbreak events in the absence of epidemiological linkage and a predefined genomic-distance threshold (Gröschel et al., 2020). The distribution of major STs also differed among human-, environmental-, and animal-derived isolates, suggesting that some lineages may be disproportionately represented in particular sources. However, these patterns may be affected by the unequal sizes of the source groups, geographic sampling, and lineage-specific sequencing or deposition practices. Moreover, differences in lineage composition may partly account for some of the observed source-associated ARG and VAG distributions. Larger and more balanced collections, together with multivariable or phylogenetically informed analyses, will therefore be required to determine whether individual lineages are genuinely associated with particular ecological reservoirs (Mercier-Darty et al., 2020).

Comparison of ARG and VAG profiles among major STs further revealed both shared and lineage-specific patterns. The broadly distributed genes likely represent common components of the resistance and virulence backgrounds of dominant *S. maltophilia* lineages, whereas differences in lower-frequency genes may reflect lineage history, ecological exposure, antimicrobial selection, and horizontal gene acquisition (Yu et al., 2016; Li et al., 2023; Yinsai et al., 2023). The uneven distribution of selected ARGs and VAGs suggests that resistance potential and VAG content are not uniform across the population and may be influenced partly by lineage background (Wang et al., 2024). Consequently, ST-level and broader phylogenetic context should be considered when interpreting the adaptive potential, epidemiological significance, and possible clinical relevance of individual isolates (Shimizu et al., 2021; Mojica et al., 2022; Wang et al., 2024).

The MGE analysis identified predicted sequence-level co-localization between ARGs and several classes of MGEs. Predicted plasmid-associated sequences constituted the largest predicted category among the ARG–MGE associations, and *blaL1* and *aph(3*’*)-IIc* were the ARGs most frequently detected on contigs classified as plasmid-associated (Joannard and Sanchez-Cid, 2024). However, the plasmid-associated results were based on a broad union criterion, whereby a contig was retained when either geNomad or MOB-suite predicted a plasmid origin; therefore, not all of the 898 plasmid-associated events should be regarded as high-confidence assignments. Concordant predictions from both tools provide stronger computational support, whereas single-tool predictions remain more uncertain. Moreover, same-contig localization indicates sequence-level association but does not establish that an ARG is carried by a complete plasmid, belongs to an intact mobile element, or is mobilizable or horizontally transferable. This limitation is particularly relevant because only 69 of the 1,584 genomes were represented by a single contig, whereas the remaining 1,515 were fragmented draft assemblies; even single-contig status does not necessarily indicate confirmed genome closure or long-read sequencing. Assembly fragmentation may therefore affect plasmid–chromosome classification and prevent reconstruction of complete ARG–MGE contexts across contig boundaries. Contig-level evidence, including genome and contig identifiers, ARG and MGE coordinates, tool-specific plasmid classifications, prediction concordance, and assembly-contiguity status, is provided in Supplementary Table S10. Complete long-read assemblies, plasmid reconstruction, and experimental transfer assays will be required to confirm the genomic localization, mobility, and transferability of these ARGs (Ogbolu et al., 2024).

Pangenome and core-genome analyses captured distinct dimensions of genomic variation between ST4 and ST5. ST4 had higher proportions of strain-specific orthogroups (3.4% versus 1.3%) and unassigned genes (2.7% versus 0.8%), indicating a larger rare-gene component among the sampled genomes, whereas the proportions of shell-plus-cloud orthogroups were nearly identical between the two lineages (46.7% versus 46.2%). Thus, the observed difference was concentrated primarily in low-frequency and strain-specific gene content and does not support an unqualified conclusion that the entire ST4 accessory genome was more diverse. ST5 had a higher proportion of orthogroups ( ≥ 99% genome occupancy) (52.4% versus 49.8%) and a higher orthogroup-assignment rate (99.2% versus 97.3%), but these occupancy and assignment metrics should not be interpreted as evidence of globally greater pangenome conservation. In contrast, the core-genome sequence analyses showed a higher pre-recombination variable-site density, more SNP sites retained after recombination filtering, and a greater median pairwise SNP distance in ST5, indicating greater sequence separation within the analyzed ST5 core-genome alignment. A larger proportion of the initially identified variable sites was also removed from ST5 as occurring within regions predicted to be affected by homologous recombination (61.1% versus 52.5%), indicating a greater predicted recombinant contribution to the unfiltered ST5 variable-site set, although this does not by itself establish a higher lineage-wide recombination rate. Taken together, these findings show that rare gene-content variation, orthogroup occupancy, and core-genome sequence divergence represent distinct genomic properties, and neither lineage should be characterized globally as “more diverse” or “more conserved” without specifying the metric being evaluated.

This study has several limitations. First, the genomes analyzed were retrieved from public databases, and the sampling distribution was uneven across countries, years, sources, and hosts. Human-derived isolates were overrepresented, whereas animal- and environment-derived isolates were relatively limited, which may affect source-associated comparisons. Second, many genomes were draft assemblies, which may limit the accurate reconstruction of plasmids, MGEs, and ARG genomic contexts. Third, the presence of ARGs and VAGs was inferred from sequence similarity, and phenotypic antimicrobial susceptibility or experimental virulence data were not available for most isolates. Despite these limitations, this study provides a comprehensive genomic overview of global *S. maltophilia* populations and integrates taxonomic classification, resistome and virulome profiling, ST distribution, source-associated comparison, MGE annotation, pangenome analysis, and SNP-based phylogeny.

## 5 Conclusion

Overall, our findings indicate that *S. maltophilia* is a globally distributed species exhibiting substantial genomic variation, conserved intrinsic resistance mechanisms, widely distributed virulence-associated traits, and variable accessory resistance determinants. The broad distribution of efflux pump-associated genes and *bla*L1 highlights the importance of intrinsic resistance, while acquired carbapenemase genes and predicted ARG-MGE co-localization highlight targets for genomic surveillance but do not establish mobilization or transferability. Source-and ST-associated differences further indicate that ecological niche and lineage background may shape the distribution of selected ARGs and VAGs. These results provide a genomic framework for understanding the evolution, adaptation, and resistance potential of *S. maltophilia* and support the need for continued genomic surveillance across clinical, animal, and environmental reservoirs.

## Data Availability

Publicly available datasets were analyzed in this study. This data can be found at: The data analyzed in this study were obtained from the following public databases: NCBI GenBank: https://www.ncbi.nlm.nih.gov/genome; NCBI BioSample: https://www.ncbi.nlm.nih.gov/biosample; VFDB: http://www.mgc.ac.cn/VFs/download.htm.

## Footnotes

1
http://www.mgc.ac.cn/VFs/download.htm

2
https://pubmlst.org/organisms/stenotrophomonas-maltophilia
